# Analysis of gene expression in a developmental context emphasizes distinct biological leitmotifs in human cancers

**DOI:** 10.1186/gb-2008-9-7-r108

**Published:** 2008-07-08

**Authors:** Kamila Naxerova, Carol J Bult, Anne Peaston, Karen Fancher, Barbara B Knowles, Simon Kasif, Isaac S Kohane

**Affiliations:** 1 Children's Hospital Informatics Program, Harvard-MIT Division of Health Sciences and Technology, Longwood Avenue, Boston, MA 02115, USA; 2 The Jackson Laboratory, Main Street, Bar Harbor, ME 04609, USA; 3 Department of Biomedical Engineering, Boston University, Cummington Street, Boston, MA 02215, USA

## Abstract

**Background:**

In recent years, the molecular underpinnings of the long-observed resemblance between neoplastic and immature tissue have begun to emerge. Genome-wide transcriptional profiling has revealed similar gene expression signatures in several tumor types and early developmental stages of their tissue of origin. However, it remains unclear whether such a relationship is a universal feature of malignancy, whether heterogeneities exist in the developmental component of different tumor types and to which degree the resemblance between cancer and development is a tissue-specific phenomenon.

**Results:**

We defined a developmental landscape by summarizing the main features of ten developmental time courses and projected gene expression from a variety of human tumor types onto this landscape. This comparison demonstrates a clear imprint of developmental gene expression in a wide range of tumors and with respect to different, even non-cognate developmental backgrounds. Our analysis reveals three classes of cancers with developmentally distinct transcriptional patterns. We characterize the biological processes dominating these classes and validate the class distinction with respect to a new time series of murine embryonic lung development. Finally, we identify a set of genes that are upregulated in most cancers and we show that this signature is active in early development.

**Conclusion:**

This systematic and quantitative overview of the relationship between the neoplastic and developmental transcriptome spanning dozens of tissues provides a reliable outline of global trends in cancer gene expression, reveals potentially clinically relevant differences in the gene expression of different cancer types and represents a reference framework for interpretation of smaller-scale functional studies.

## Abstract 2

A systematic analysis of the relationship between the neoplastic and developmental transcriptome provides an outline of global trends in cancer gene expression.

## Background

The historical roots of our understanding of the intimate connection between tumorigenesis and developmental processes reach back to 1858, when Rudolf Virchow first suggested that neoplasms arise "in accordance with the same law, which regulates embryonic development" [1]. Since then, his idea has profoundly influenced medicine and still remains highly relevant today. The similarities between cancer and development are evident on many levels of observation: microscopically, cancerous tissues appear as undifferentiated masses, with some tumor types even exhibiting embryonic tissue organization. The increased mobility of malignant cells, leading to invasion of the local environment with the potential for subsequent travel to distant organs (representing one of the most problematic clinical aspects of cancer), is reminiscent of migratory behavior during development. On the molecular level, the shared characteristics between certain malignant tumors and developing tissues with respect to transcription factor activity [2], regulation of chromatin structure [3] and signaling [4] have been documented. In particular, several studies have suggested that part of the cancer transcriptome represents a 'developmental signature', that is, it contains a set of genes that are collectively active during development. For lung cancer [5,6], liver cancer [7], Wilms' tumor [8], colon cancer [9,10] and medulloblastoma [11], gene expression patterns resembling early developmental stages of the corresponding organ have been identified in the tumor profile. The results of these transcriptome-scale analyses are important because they offer a glimpse into fundamental biological processes underlying tumorigenesis and provide a natural framework for understanding complex cancer gene expression signatures that are difficult to interpret otherwise. Moreover, developmental signatures harbor a clinical relevance that we are only beginning to discover. For example, lung cancers can be risk-stratified by their similarity to lung development and pluripotency gene signatures can be used to predict outcome in breast cancer [6,12].

In the present study, we paint a novel picture of the oncological landscape by comparing a variety of human cancers based on their developmental signature. Our analysis was inspired by the following questions: to which extent can the transcriptome of a tumor, which is oftentimes perceived as an aberration, be 'explained' by developmental gene expression? Does the developmental signature represent a feature of most, and possibly all, human cancers or does gene expression in different tumors fall into distinct groups with respect to development? Is recapitulation of developmental gene expression programs a tissue-specific phenomenon or is the developmental signature largely composed of general transcriptional modules that play a ubiquitous role in developmental processes? The answers to these open questions have therapeutic implications [13]. If a broad range of tumors employs primitive developmental mechanisms that are shared across tissues to sustain their growth and survival, a certain drug or class of drugs could be capable of affecting them all. If, on the other hand, highly lineage-specific mechanisms govern malignant growth and behavior, focus has to be put on identifying and targeting tissue-specific regulators.

The results from the integrative analysis of gene expression in cancer and development presented here suggest that the developmental information content of most human cancers indeed is significant. The developmental signature of cancers originating from various tissues exhibits low tissue-specificity, indicating that a large portion of the cancer transcriptome is composed of general developmental modules. Furthermore, we describe three developmentally distinct groups of cancer, validate the class distinction on a new time series of embryonic development in the mouse and show that the behavior of genes in lung development is predictable by their expression across the three groups. We explore the biological themes dominating the expression profiles of these classes and demonstrate that one group recapitulates early developmental gene expression patterns and is characterized by an 'individualistic' signature with upregulation of pluripotency genes and suppression of genes involved in cell-cell communication and signal transduction. A second group exhibits a 'communicative' gene expression signature that is active in late development, is enriched in genes involved in immune response, cell-cell and cell-matrix interactions and resembles a wound healing signature. A third group connects the previous two with a transition phenotype. While social and anti-social aspects of cancer have been widely popularized, this study points out the possibility of a more subtle classification of different cancers that tend to evoke different types of 'survival mechanisms'. Finally, we identify a core program of genes that are upregulated in most cancers and show that these genes are coexpressed in early development.

## Results

### Placing human cancers on a developmental landscape

Our analysis is based on a large-scale comparison of gene expression in 10 developmental processes and 32 cancer data sets. To paint an unbiased picture of the association between gene expression in development and oncogenesis, we selected data from a wide biological range. Our development database encompasses gene expression time series characterizing processes as diverse as heart development in the mouse, human T cell development and *in vitro *differentiation of murine embryonic stem cells (see Additional data file 6 for a list of all data sets). Cancer gene expression data include tumors from most commonly affected anatomical locations and corresponding normal tissue as a reference.

**Figure 1:**
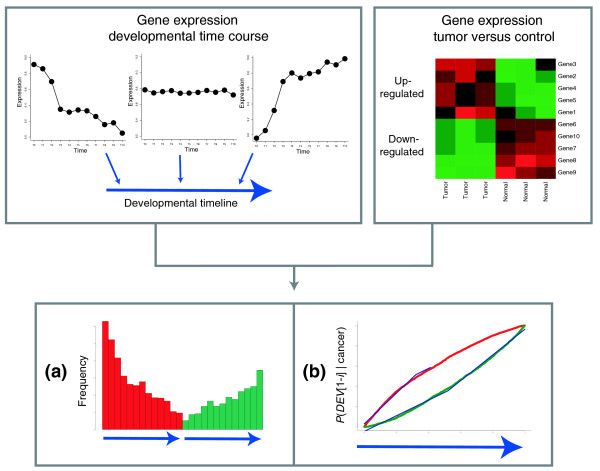
Approach to data analysis. A developmental timeline (DT), which is a linear number ray on which each of 5,166 genes has a definite position, is constructed from a time course of gene expression during development (top left panel), positioning genes that are expressed in early development on the left end, genes that are upregulated in late development on the right end and neutral genes in the middle. The DT is integrated with genes that are deregulated in a population of tumors versus corresponding normal tissues (top right panel). **(a) **Frequency plot showing a histogram-like representation of the frequency of upregulated (red) and downregulated (green) cancer genes in different portions of the DT. The height of each bar indicates how many deregulated genes map to one of 13 equally sized segments of the DT. Each segment corresponds to approximately 400 genes. Up- and downregulated genes are depicted on separate DTs, that is, the first red bar refers to the same DT segment as the first green bar. Stated differently, the height of the first red bar signifies the number of upregulated cancer genes that map to the first 400 developmental genes and the height of the first green bar signifies the number of downregulated cancer genes that map to the same set of 400 developmental genes. **(b) **Probability density plot showing *P*(*DEV*[*1*,*2*,*3...i*]* | cancer*) for *i *= 2,3...5,166 for upregulated and downregulated cancer genes. The probability of being among the first *i *genes on the DT (genes are numbered 1-5,166 from left/early to right/late) if deregulated in cancer directly reflects the preference of cancer genes for different segments of the DT. The shape of each probability distribution is summarized by two linear functions that are fitted to its early and late portions (blue lines). The slopes of these functions are subsequently used as a quantification of the developmental profile of a cancer.

The approach for analysis of this large data compendium (consisting of 1,094 individual arrays) is depicted in Figure 1. We first simplified the complex, high-dimensional expression profiles characterizing each developmental process into a one-dimensional developmental timeline (DT). To understand the DT, it is necessary to first consider some general properties of gene expression dynamics during a continuous developmental process: starting at the earliest (least differentiated) instance of a series of conditions, genes that are characteristic of an immature state will be active. As development progresses, the expression of these genes will gradually abate. Concomitantly, the expression of genes that are specific for the mature state will continuously intensify until it reaches its peak at the latest (most differentiated) point in time. On average, about 30% of the measured genes will follow this pattern. The construction of the DT takes advantage of this behavior, ordering the genes in a linear array based on their temporal pattern of expression. Early genes are localized on the left end of the DT, genes with no bias towards early or late expression center in the middle and late genes occupy the right end. Thus, the unique order of genes on the DT represents a summary of early and late states for each developmental process.

In the next step, we determined the relationship of gene expression in cancer to each of the ten DTs. We identified the genes that were up- and downregulated in a cancer relative to its corresponding normal tissue and tracked their position (or the position of their mouse ortholog for murine developmental processes) on the DTs [11]. In the following, we will use two kinds of plots to summarize the resulting distribution: a frequency plot (Figure 1a) for an intuitive overview of where deregulated cancer genes fall on the DT and a probability density plot (Figure 1b) that allows a more accurate quantification of the cancer-development relationship. The frequency plot is divided into two panels: on the left side, the frequency of upregulated genes on the DT is shown; on the right side, the DT is depicted again with the distribution of downregulated genes (Figure 1).

The probability density plot shows how likely genes in different segments of the DT are to be expressed/suppressed in cancer (see the Figure 1 legend for details). If there was no correlation between gene expression in cancer and development, the probability distributions would follow a straight line with slope 1. However, if certain parts of the DT contain genes that are up- or downregulated in cancer with a higher frequency than expected by chance, the slope of the probability density increases. Conversely, if cancer genes are depleted in a particular segment of the DT, the slope becomes flatter. For the deregulated genes in Figure 1b, this results in an 'open eye' shape of the probability density (the legend to Figure 1 details the quantification of this shape).

### A variety of cancers have activated a predominantly tissue-independent developmental signature

**Figure 2:**
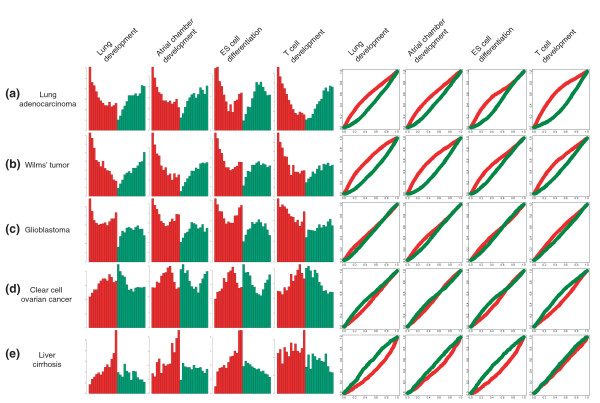
Frequency plots and probability distributions for **(a) **lung adenocarcinoma, **(b) **Wilms' tumor, **(c) **glioblastoma, **(d) **clear cell ovarian cancer and **(e) **liver cirrhosis. These cases were selected because they are representative of most tumors in our database.

We will discuss some general principles emerging from the comparison of all our data sets to the ten DTs on a subset of instances and progress to a global overview thereafter. Figure 2 shows the frequency plots and probability distributions for lung adenocarcinoma, Wilms' tumor, glioblastoma, ovarian cancer and liver cirrhosis with respect to the DTs of lung development, atrial chamber development, embryonic stem (ES) cell differentiation and T cell development. The distribution of lung adenocarcinoma genes on the lung development DT represents a good starting point for discussion, given that the recapitulation of embryonal pulmonary gene expression in lung cancer has been reported repeatedly [4,5]. The frequency plot shows an early peak for upregulated genes, followed by a gradual decline towards the late end of the DT, implying that genes that are active in lung adenocarcinoma are preferentially expressed in early lung development. The pattern is inversed for downregulated genes, meaning that genes that are characteristic for the mature, differentiated state of the lung are suppressed in lung cancer. The probability density confirms this observation with a sharp rise of *P*(*DEV*[1-*i*] | *cancer*) for low values of *i* (early development) for upregulated genes and high values of *i* (late development) for downregulated genes.

Perhaps unexpectedly, the specificity of upregulated lung cancer genes for early development (and downregulated genes for late development) can be reproduced on DTs derived from atrial chamber development, ES cell differentiation and T cell development (more examples can be found in Additional data file 1). Apparently, gene expression programs that are exploited during lung tumorigenesis play a ubiquitous role in processes involving differentiation and morphogenesis. This result is in contrast to the prevailing notion that recapitulation of developmental gene expression in cancer is a tissue-specific phenomenon [9,11].

Examination of the developmental distribution of Wilms' tumor genes suggests that this property is not unique to lung cancers. The segregation of up- and downregulated genes in Wilms' tumor on lung development occurs even more convincingly than the separation of lung cancer genes. A similar result for many other tumor types (Additional data file 1) suggests that this is unlikely to be solely attributable to the embryonal nature of Wilms' tumor. Instead, a general developmental signature that shows very little evidence of tissue-specificity seems to be a hallmark of many cancers. However, there are several notable exceptions.

Upregulated genes in glioblastoma (2c) follow a similar pattern to lung adenocarcinoma and Wilms' tumor in early development, but an additional peak prominently occurs on the late end of the DTs. Beyond expressing early genes, glioblastomas have activated other, distinct transcriptional programs that are characteristic of later developmental stages. The developmental gradient in this case is not capable of 'explaining' the glioblastoma gene expression signature unambiguously. An even more striking example is ovarian cancer (Figure 2d), a tumor that is in many respects the developmental complement of glioblastoma: upregulated genes tend to avoid early and late development, while downregulated genes have a preference for the extremes of the DT. Apparently, transcriptional states in different cancers map to distinct domains of physiological gene expression. These divergent developmental patterns are unlikely to be random fluctuations. First, their recurrence with respect to changing developmental backgrounds suggests a robust association. Second, up- and downregulated genes have complementary patterns; where upregulated genes are abundant on the DT, downregulated genes are infrequent and vice versa. The expression of certain sets of genes seems to be mutually exclusive; if one set is active, the other set is invariably turned off. Third, a limited number of patterns consistently recurs in different data sets.

Finally, Figure 2e shows the developmental profile of a disease that does not directly belong to the cancer family: liver cirrhosis. The developmental timing of deregulated genes in cirrhosis is strikingly different from most cancers. Upregulated genes have a preference for late development, downregulated genes tend to be enriched on the early end of the DTs. This example illustrates that the distribution of deregulated genes in development indeed is a pathophysiology-specific phenomenon.

### Three distinct groups of tumors emerge from the developmental landscape

The cases discussed in Figure 2 are a collection of representative examples highlighting some fundamental properties of the association between cancer and development. By visual inspection it is already clear that the developmental profiles of lung adenocarcinoma and Wilms' tumor are more similar to each other than to ovarian cancer, for example. However, if we want to extend this assessment of similarity to a larger number of tumors, a quantitative description of the 'shape' of the developmental profile is required. We realized this quantification by fitting two linear curves to each probability distribution, one curve representing its slope in the early part of the DT and the other one approximating the late slope (Figure 1b). Thus, each combination of cancer and developmental process is summarized by a unique set of four values, consisting of two slopes for upregulated and two slopes for downregulated genes.

**Figure 3:**
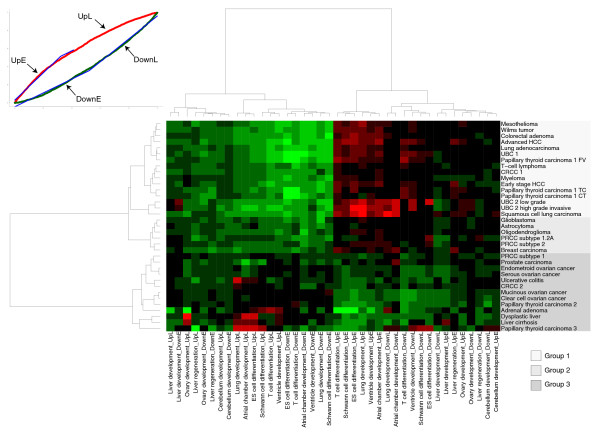
Heatmap of probability distribution slopes. Thirty-two expression data sets of neoplasia versus corresponding normal tissue (and liver cirrhosis versus normal liver, dysplastic liver versus normal liver and ulcerative colitis versus non-inflamed colon) are compared against all 10 DTs. Each comparison is characterized by a four-dimensional vector of slopes derived from the probability distributions (example in top left corner). Two slope values stem from the distribution of upregulated genes on the DT, two are derived from the distribution of downregulated genes (Figure 1). UpE = slope for upregulated genes in the early part of the DT; UpL = slope for upregulated genes in the late part of the DT; DownE = slope for downregulated genes in the early part of the DT; DownL = slope for downregulated genes in the late part of the DT. Red indicates a steep slope (high specificity of up- or downregulated genes for that segment of the DT), green indicates a flat slope (depletion of up- or downregulated genes in that segment).

We next used this set of values to establish a high-level overview of the developmental information in all our datasets. Clustering by the probability distribution slope values (Figure 3) reveals at least three distinct groups of tumors that exhibit disparate developmental patterns. Group 1 contains tumors with 'early' developmental profiles comparable to lung adenocarcinoma and Wilms' tumor (Figure 2). This group represents 46% of all datasets and contains tumors from a diversity of anatomical locations, including lung carcinomas, bladder cancers, hepatocellular carcinomas and the hematological malignancy T-cell lymphoma. Clearly, early developmental gene expression is a widespread feature in cancer. An important observation is that the early developmental signature in all these tumors is only minimally tissue-specific. Many cancers have approximately equal slope values across diverse developmental backgrounds, meaning that deregulated genes map with the same specificity to the early and late segments of many DTs.

Group 2 contains several tumors with an ambiguous correlation with developmental gene expression. Glioblastoma is part of this group, next to several other central nervous system tumors, breast cancer, and the more aggressive forms of papillary renal cell carcinoma (subtypes 1.2A and 2). Examination of the frequency plots and probability distributions for these cancers (Additional data file 1) shows that two types of tumors are found in this group: those that do recapitulate early developmental gene expression, but also exhibit additional transcriptional programs that are not consistent with the developmental gradient (for example, glioblastoma); and tumors that are consistent with the gradient, but whose deregulated genes show a less dramatic preference for the extremes of the DTs (for example, breast carcinoma).

Group 3, featuring several subtypes of ovarian cancer, prostate cancer, two independent data sets of papillary thyroid carcinoma (PTC) and two independent instances of renal cell carcinoma, displays a transcriptional phenotype that is completely distinct from groups 1 and 2. Upregulated genes have no clear preference for early development. In fact, in some instances they accumulate on the late end of the DTs, co-clustering with liver cirrhosis, dysplastic liver and ulcerative colitis. The behavior of downregulated genes varies considerably. In some cases - most notably the ovarian cancers - they complement upregulated genes, but in PTC 3 for example, up- and downregulated genes peak in similar DT segments, hinting at active regulatory mechanisms that are not found in normal developmental processes. It is apparent that group 3 is a much more heterogeneous collection of diseases than groups 1 or 2.

Of note, two data sets in group 3 have counterparts of histologically similar tumors located in group 1. PTC is represented with three, and clear cell renal cell carcinoma (CRCC) with two independent data sets in our database. Two of the PTC data sets belong to group 3; a third data set, which is divided in three histological subtypes of PTC (follicular, tall cell and conventional variant) is part of group 1. Possibly, the lacking histological subclassification of PTCs belonging to group 3 emphasizes a different transcriptional theme in those tumors. Even more likely, the paired experimental design of the two group 3 PTC data sets - in both cases, tissue from the same patient served as a normal control - influences the gene expression signature. We will address this issue in more detail in the discussion.

The CRCC data sets are concordant as far as the top third of differentially expressed genes is concerned. Considering only the 450 most differentially expressed genes reveals a pronounced preference of upregulated genes for the late part of DTs in both data sets (Additional data file 3), making CRCC more similar to diseases like liver cirrhosis and ulcerative colitis and implying that the early peak that places CRCC 1 among the 'early developmental' tumors is a less significant addition to a prominent 'late' transcriptional program.

While groups 1 and 3 are clearly distinct, it is debatable whether group 2 should be treated as its own entity. It is apparent that there is a spectrum of developmental signatures, with most cancer types clustering at its early or late end and a few intermediate cases that cannot be classified unambiguously. Examining the distribution of probability distribution slope values for upregulated genes in the early segment of the DTs (the most distinguishing feature) exemplifies this point (Additional data file 8). The distribution is bimodal, with most cancers falling into the early or late peak and group 2 tumors occupying the middle. To achieve a clear biological separation in subsequent analyses, we decided to treat these intermediate cases as a distinct class; it remains to be determined in more comprehensive studies whether this group can be identified reproducibly.

### The contribution of proliferation-related genes to the developmental pattern in cancer

Since early stages of most developmental processes involve massive proliferation, part of the similarity between early development and cancer can most certainly be attributed to cell cycle (CC)-related genes. Also, the clinical behavior of the cancers constituting the three groups raises the question whether a proliferation signature could be driving their developmental profile. Group 1 mostly consists of aggressive tumors with low doubling times (for example, urinary bladder cancer, lung cancer, Wilms' tumor), while group 3 contains more indolent forms. Tumors like ovarian and renal cancer are associated with poor outcome because they metastasize frequently and do not respond well to chemotherapy, but their growth rate tends to be relatively low [14-16]. Also, prostate and thyroid cancers are well-known for their slow growth [17,18].

**Figure 4:**
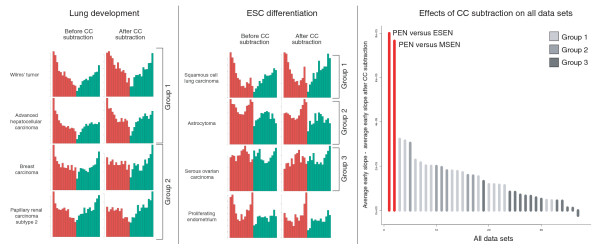
Effects of CC subtraction. Frequency plots of selected cancer types on the backdrop of lung development (left panel) and ES cell differentiation (middle panel) are depicted before and after the dismissal of hundreds of CC regulated genes. The corresponding probability distributions can be viewed in Additional data files 9 and 10. The right panel shows the effects of this CC subtraction on all data sets, quantified as the difference of the early probability distribution slope value (UpE) before and after elimination of CC regulated genes. PEN versus ESEN = proliferating endometrium versus early secretory endometrium; PEN versus MSEN = proliferating endometrium versus mid secretory endometrium.

In order to determine whether the developmental component in cancer is more than a proliferation signature, we rigorously eliminated genes that are correlated with progression through the CC in HeLa cells [19] from the deregulated genes of all cancers (see Materials and methods), discounting approximately 50% of differentially expressed genes in many data sets. Figure 4 shows selected developmental profiles before and after this CC subtraction. Group 1 tumors are largely unaffected. Their profiles become noisier due to the reduction of the number of differentially expressed genes, but the shape remains qualitatively unchanged. In group 2, however, the early peaks in the frequency distribution disappear, suggesting that the CC is a dominant factor in the upregulated genes mapping to early development here, which does not seem to be the case in group 1. The profiles of group 3 tumors also remain constant. To see whether this surprising robustness to CC subtraction is a cancer-specific phenomenon, we constructed a developmental profile for proliferating endometrium (PEN) versus early secretory endometrium (ESEN) as a model for a proliferating, but non-malignant tissue. Similarly to tumors in group 1, most genes upregulated in PEN map to early development. In contrast to cancer, however, the effects of CC subtraction are much more pronounced. Figure 4c shows a quantitative assessment of these effects, defined as the difference of the probability density slope for early upregulated genes before and after CC subtraction. Clearly, the developmental component in cancer is less CC dominated than in the PEN. This becomes particularly visible on the background of ES cell differentiation (Figure 4b). Discounting CC-regulated genes completely eliminates the early peak in the frequency distribution for PEN, while the profile for squamous cell lung carcinoma and other group 1 tumors (Additional data file 2) does not change. This demonstrates that cancer shares a common gene expression signature with stem cells that cannot be found in normal PEN tissue. Finally, clustering all data sets by their probability distribution slope values after CC subtraction results in the same distinction between groups 1, 2 and 3 as the one shown in Figure 3 (Additional data file 4). We therefore conclude that the CC is not the main determinant of the disparate gene expression programs in these tumors.

### Gene expression in groups 1, 2 and 3 is dominated by different biological processes

We next used Gene Ontology (GO) to compare the dominant biological processes in groups 1, 2 and 3 with two developmental meta-signatures, eDEV500 and lDEV500, representing tissue-independent early and late programs. eDEV500 is defined as the 500 genes that are most consistently expressed early across all time series (analogous definition for lDEV500). Table 1 shows that upregulated genes in groups 1 and 2 are enriched for the same processes as eDEV500, most prominently CC, RNA splicing and DNA repair. Indeed, DNA repair genes are active in pre-implantation and late gestational development and have been shown to be essential for embryonic viability and development of extra-embryonic tissues [20]. Downregulated genes in group 1 belong to processes that are underrepresented in eDEV500 and enriched in lDEV500. These include cell communication, signal transduction and system development, processes that are required for the establishment and maintenance of a structured tissue organization. It is noteworthy that downregulated genes in group 2 diverge from this theme. The prominent observation here is that genes required for aerobic respiration are reduced; this could either point at hypoxic conditions or the Warburg effect (a shift towards lactate production in cancer cells even under normal oxygen supply). From a developmental perspective, upregulated genes in group 3 represent a mirror image of group 1. They map to similar terms as lDEV500, namely immune response, cell adhesion and multicellular organismal process. While the latter two processes clearly gain importance in the course of organogenesis, immune response is less obviously associated with late developmental stages. The role of cytokine signaling in hematopoiesis is well-established, but its function in the development of other tissues is incompletely understood. However, it is becoming clear that chemokines do not only function as chemoattractants for immune cells during inflammation, but also fulfill essential roles in embryogenesis and tissue homeostasis [21]. For example, inhibition of signaling through the chemokine receptor CXCR4 leads to defects in migration and differentiation in the developing chick limb [22]. In cancer, chemokine signaling can also affect migratory behavior. For instance, mesenchymal stem cells in the tumor stroma are able to increase breast cancer cell motility through paracrine CCL5 signaling [23]. The expression of inflammation-related genes in cancer tissue is frequently interpreted as a consequence of an immune response against the tumor. Interestingly, the developmental perspective suggests that a similar gene expression signature exists during the normal development of several tissues without the involvement of an inflammatory reaction.

**Table 1:** GO category enrichment

	BP - overrepresented	BP - underrepresented	CC - overrepresented
eDEV500	DNA replication Cell cycle RNA splicing DNA repair Chromatin modification	Multicellular organismal process Cell communication Signal transduction System development Ion transport	Intracellular Nuclear part Membrane-bound organelle Spliceosome Ribonucleoprotein complex
			
lDEV500	Immune response Antigen processing and presentation Cytokine and chemokine mediated signaling pathway Cell adhesion Multicellular organismal process	Biopolymer metabolic process Biosynthetic process RNA processing Cell cycle phase DNA repair	Membrane Extracellular region MHC protein complex Lysosome Secretory granule
			
Group 1 (16)			
Up	DNA repair (15) Cell cycle (15) RNA splicing (13)	Multicellular organismal process (16) G-protein coupled receptor protein signaling pathway (16) Neurological process (16)	Intracellular (16) Organelle (15) Nuclear part (15)
Down	Multicellular organismal process (15) Organ development (14) Cell communication (11)	Primary metabolic process (14) RNA processing (14) DNA metabolic process (14)	Plasma membrane (16) Extracellular region (13) Voltage-gated potassium channel complex (8)
			
Group 2 (6)			
Up	Cell cycle (6) DNA replication (6) Response to DNA damage stimulus (6)	Multicellular organismal development (5) Anatomical structure development (5) System development (4)	Chromosome (6) Protein complex (5) Replication fork (5)
Down	Monovalent inorganic cation transport (5) ATP synthesis coupled proton transport (5) Oxidative phosphorylation (4)	DNA recombination (6) Immune response (5) Macromolecule metabolic process (5)	Proton-transporting two-sector ATPase complex (5) Membrane (5) Extracellular matrix (3)
			
Group 3 (13)			
Up	Immune response (10) Multicellular organismal process (8) Cell adhesion (6) Response to wounding (5)	Cellular metabolic process (10) Nucleobase, nucleoside, nucleotide and nucleic acid metabolic process (9) RNA metabolic process (8)	Plasma membrane (10) Extracellular region (10) Lysosome (5)
Down	Cellular metabolic process (10) Protein metabolic process (6) RNA processing (5)	Multicellular organismal process (10) Immune response (10) Cell activation (8)	Cytoplasm (10) Intracellular (8) Organelle (8)
			
PEN versus ESEN			
Up	DNA replication Cell cycle phase DNA metabolic process	Biosynthetic process Generation of precursor metabolites and energy Translation	Chromosome Replication fork Microtubule cytoskeleton
Down	Lipid metabolic process Lipid biosynthetic process Cofactor metabolic process	Macromolecule metabolic process Intracellular signaling cascade M phase of mitotic cell cycle	Desmosome Membrane fraction Microsome

Next to the most significant GO categories for eDEV500, lDEV500 and PEN versus ESEN, the GO categories that are most frequently enriched in the up- and downregulated genes of group 1, 2 and 3 data sets are listed with the number of occurrences in parentheses. BP, biological process; CC, cellular component. For example, DNA repair is enriched in the upregulated genes of 15 out of 16 data sets belonging to group 1.

The difference between early and late developmental genes, and consequently genes activated in group 1 versus group 3, is also evident when comparing the cellular localization of their gene products. Proteins that are produced in early development and group 1 are predominantly located in the nucleus. Similarly, upregulated genes in group 2 have products with nuclear localization and specific involvement in the CC. Gene products of lDEV500 and group 3, however, are chiefly membrane-associated or secreted into the extracellular space.

Finally, we compared the PEN to development and cancer. As expected, upregulated genes were mostly CC-related. However, they were not depleted for cell communication or signal transduction genes like eDEV500 and cancers in groups 1 and 2, suggesting that proliferating cells of the endometrium retain a higher level of communication with their surroundings than those in cancer or early development. Downregulated genes were associated with lipid metabolism and showed no enrichment for organogenesis or multicellular processes like lDEV500 and downregulated genes in group 1. Taken together, these results suggest a unique relationship between malignancy and development that is not fully recapitulated in normal proliferating tissues.

### Among hundreds of curated gene sets, the developmental signature is the best descriptor of approximately 50% of interrogated tumor types

We next wanted to determine how well our developmental signatures describe the difference between cancer and normal tissue in a direct comparison with other gene sets. We downloaded the C2 database from MSigDB [24], a collection of gene sets derived from gene expression studies and known pathways, and tested the enrichment of approximately 1,000 gene sets in the up- and downregulated genes of our data sets. Subsequently, we compared the results with the performance of eDEV500, lDEV500 and four smaller gene sets that were defined analogously, eDEV200/lDEV200 and eDEV100/lDEV100.

Table 2 shows the gene sets that were most significantly enriched in the up- and downregulated genes of the three groups. Upregulated genes in group 1 are best represented by eDEV500, which is a remarkable result because no cancer gene expression data were used in deriving this gene set, but solely time courses of mouse development (all DTs except for T cell development are murine). Many data sets in MSigDB, on the other hand, are directly derived from gene expression profiles of human cancers. Of course, the groups were defined by the distribution of deregulated genes in development, but group 1 is not a specialized subset, but comprises almost 50% of our data sets. Two of the top ranks next to eDEV500 and eDEV200 are occupied by sets of genes that are upregulated in stem cells, implying a close connection between early development and pluripotency that is also evident in the cancer gene expression profile. CC gene sets are not among the most enriched signatures, but the imprint of 'stemness' can clearly be distinguished in group 1 tumors, even though our data sets represent heterogeneous tissues containing a variety of cell types. Conversely, lDEV500 is the most significant gene set in the downregulated genes of group 1, next to genes that are downregulated in various tumor models (SANSOM_APC_5_DN, LEE_DENA_DN, LEE_ACOX1_DN) and signatures found in activated mast cells (NAKAJIMA_MCS_UP), confirming the aforementioned association of late developmental genes and downregulated genes in group 1 cancers with the immune response.

**Table 2:** MSigDB C2 gene sets most significantly enriched in groups 1-3

Upregulated genes	Downregulated genes
Group 1	
eDEV500	lDEV500
STEMCELL_NEURAL_UP	SANSOM_APC_5_DN
eDEV200	NAKAJIMA_MCS_UP
TARTE_PLASMA_BLASTIC	TARTE_MATURE_PC
STEMCELL_EMBRYONIC_UP	CALCIUM_REGULATION_IN_CARDIAC_CELLS
PRMT5_KD_UP	LEE_DENA_DN
CANCER_NEOPLASTIC_META_UP	SMOOTH_MUSCLE_CONTRACTION
LI_FETAL_VS_WT_KIDNEY_DN	YAO_P4_KO_VS_WT_UP
eDEV100	lDEV200
MOREAUX_TACI_HI_IN_PPC_UP	LEE_ACOX1_DN
	
Group 2	
HOFFMANN_BIVSBII_BI_TABLE2	FLECHNER_KIDNEY_TRANSPL_REJ_DN
YU_CMYC_UP	AGEING_KIDNEY_SPECIFIC_DN
DNA_REPLICATION_REACTOME	CHANG_SERUM_RESPONSE_DN
eDEV500	LE_MYELIN_DN
SERUM_FIBROBLAST_CORE_UP	AGEING_KIDNEY_DN
CMV_IE86_UP	VENTRICLES_UP
CHANG_SERUM_RESPONSE_UP	CARIES_PULP_DN
G1_TO_S_CELL_CYCLE_REACTOME	UVB_NHEK1_UP
PEART_HISTONE_DN	SMOOTH_MUSCLE_CONTRACTION
GENOTOXINS_ALL_4HRS_REG	BRCA_ER_POS
	
Group 3	
SERUM_FIBROBLAST_CELLCYCLE	FLECHNER_KIDNEY_TRANSPL_REJ_DN
BRCA_ER_NEG	AGEING_KIDNEY_DN
TARTE_MATURE_PC	IDX_TSA_UP_CLUSTER6
DAC_PANC_UP	AGEING_KIDNEY_SPECIFIC_DN
SANSOM_APC_5_DN	DIAB_NEPH_DN
NAKAJIMA_MCS_UP	CARIES_PULP_DN
CANCER_UNDIFFERENTIATED_META_UP	MITOCHONDRIA
HIF1_TARGETS	BRCA_ER_POS
LEE_TCELLS3_UP	VENTRICLES_UP
GENOTOXINS_ALL_4HRS_REG	HEARTFAILURE_ATRIA_DN

eDEV500 is less significant in group 2 than in group 1. This is consistent with previous results showing a less pronounced clustering of upregulated genes in early development for group 2. Instead, two independent serum response signatures are enriched in the upregulated genes (SERUM_FIBROBLAST_CORE_UP, CHANG_SERUM_RESPONSE_UP). Besides stimulating proliferation, serum exposure induces a wound healing response in fibroblasts, involving the activation of genes that play a role in intercellular signaling and remodeling of the extracellular matrix [25]. These are both processes that map to late development in our analysis. Indeed, group 2 tumors tend to have both an early and a late peak in the frequency distribution of upregulated genes (Figure 2).

As already noted in the context of GO classification, gene sets enriched in group 3 are a counterpart of group 1. eDEV500 does not rank among the top gene sets, nor do any of the stem cell signatures. Instead, three signatures that are enriched in group 1 downregulated genes are overrepresented in the upregulated genes of group 3 (TARTE_MATURE_PC, SANSOM_APC_5_DN, NAKAJIMA_MCS_UP). The combination of serum-induced cell division (SERUM_FIBROBLAST_CELLCYCLE) and immune response gene sets again suggests an association with wound healing, but the early developmental component that is so prominent in group 1 and also present in group 2 is lacking in group 3.

**Figure 5:**
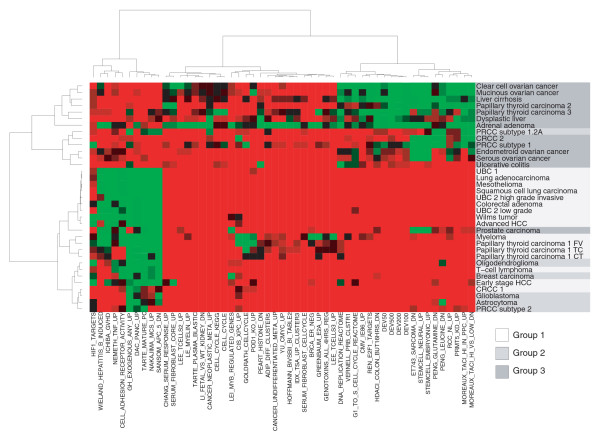
Heatmap of enrichment *p*-values. The *p*-values for gene sets that ranked among the 20 most enriched in the upregulated genes of either group 1, 2 or 3 are shown for all data sets. Red indicates low *p*-values, green high *p*-values.

To visualize how well the tumors inside of a group agree on the significance of a gene set, we clustered all data sets by the *p*-values for the top 20 signatures in the upregulated genes of the three groups (Figure 5). Group 1 presents very homogeneously with only few exceptions such as the thyroid carcinomas and renal carcinoma. Both of these cancers have counterparts in group 3 and have already been mentioned as ambiguous cases. The variation in group 2 is also low. Its position as a transition state between groups 1 and 3 is clearly visible in the heatmap as a general agreement with group 1, but simultaneous activation of a cluster of gene sets (hypoxia response, immune response, cell adhesion receptor activity) that are enriched in group 3 and insignificant in group 1. Group 3 clearly represents a distinct entity, but intra-group variation is substantial, confirming a greater heterogeneity among these tumors. Notwithstanding, they are all characterized by the lack of a pronounced developmental/stemness component and activation of inflammatory signatures. An analogous heatmap for gene sets enriched in downregulated genes (Additional data file 5) shows that the distinction of groups 1-3 is also present in genes that are suppressed in these cancers.

### The class distinction is reproducible on an independent time series

**Figure 6:**
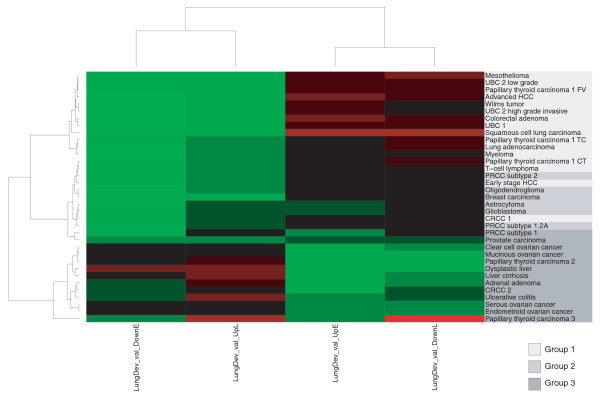
Heatmap of probability distribution slopes for all data sets with respect to the lung development validation time series. Abbreviations and colors are the same as in Figure 3.

**Figure 7:**
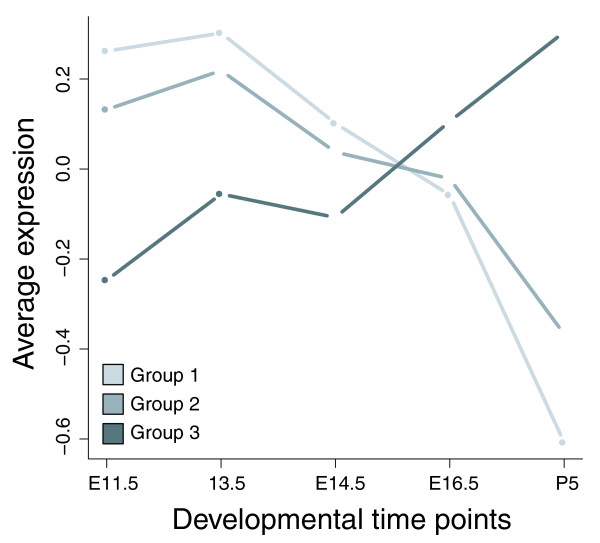
Average expression level of consensus gene sets in the lung development validation time series. Consensus group 1 = genes overexpressed in 11/16 data sets belonging to group 1; consensus group 2 = genes overexpressed in 5/6 data sets belonging to group 2; consensus group 3 = genes overexpressed in 8/13 data sets belonging to group 3.

To test whether we could validate the segregation of tumors into distinct developmental classes on an independent time series, we generated expression profiles of the developing mouse lung at embryonic day (E) 11.5, E13.5, E14.5, E16.5 and postnatal day 5. A heatmap of probability distribution slope values based on the DT constructed from these data (Figure 6) shows that the segregation of tumors into the previously defined groups can be fully recapitulated. This result further corroborates that the relationship between a cancer type and developmental gene expression is highly robust. Given that groups 1(2) and 3 display such disparate developmental patterns, we next asked whether the fact that a gene is upregulated in group 1, 2 or 3 is enough to predict its behavior during embryonic lung development. Based on our previous results, we would expect genes that are commonly upregulated in group 1 to be expressed in early lung development, group 2 genes to have a more ambiguous expression pattern and genes activated in group 3 to be expressed late. We defined three consensus signatures by selecting those genes that are expressed in at least 60% of the data sets in each group (80% for group 2 to account for smaller group size). Figure 7 shows the average expression value for the three consensus gene sets at each time point in our lung developmental time series. Indeed, consensus genes for groups 1 and 2 are upregulated in early development (with a more pronounced decline of group 1 genes in late development), while group 3 genes are active late. Remarkably, the fact that a set of genes is expressed in a particular group of tumors is enough to predict the average temporal expression pattern of these genes during embryonic development in a different species, further highlighting the deep-rooted connection between development and tumorigenesis.

Table 3 shows some examples of the consensus genes, sorted by their average rank across all DTs. Consensus genes in group 1 are almost exclusively expressed very early on (average rank <1,500) and include molecular chaperones (*CCT3*, *CCT7*), proliferation-related genes (*RACGAP1*, *PCNA *and its associated factor KIA1001 [26]) and DNA repair genes (*UNG*, *H2AFX*). The consensus set of group 2 includes genes that are expressed early and late. Early genes fall into similar categories as the group 1 consensus, that is, DNA repair (*NUDT1*, *DTL*), proliferation (*DTMYK*, *MELK*) and DNA methylation (*HELLS*). Late genes are involved in signal transduction (*DTNA*) and antigen processing (*TAP1*). *PSMB8 *and *PSMB9 *are part of the immunoproteasome, a special form of the proteasome that is active after stimulation of cells with interferon-γ and is constitutively expressed in dendritic cells. The immunoproteasome exhibits modified cleavage properties that have been shown to affect tumor antigen processing and consequently cytotoxic T cell responses [27]. Consensus genes in group 3 mainly map to late development and are involved in antigen processing (*HLA-C*, *TAP1*), extracellular matrix remodeling (*MMP7*), proteolysis (*PPGB*), and cytokine signaling (*IL7R*). However, the group 3 consensus also contains a fraction of early genes that overlap with early consensus genes in groups 1 and 2 (*CKS2*, *MELK*, KIAA0101*, *RACGAP1*). Considering the small size of the consensus gene sets - 20, 58 and 29 genes for groups 1-3, respectively - this level of unanimity is striking and suggests the existence of a 'core program' that is active in all cancers, regardless of large-scale differences in the global gene expression program.

**Table 3:** Example genes from the consensus sets of groups 1-3 ordered by their average rank across all DTs

	ProbeID	Average rank	Gene symbol	Description
Consensus group 1				
Early	201577_at	627.1	NME1*	Non-metastatic cells 1, protein (NM23A)
	200812_at	633.8	**CCT7**	Chaperonin containing TCP1, subunit 7 (eta)
	201202_at	875.7	**PCNA**	Proliferating cell nuclear antigen
	205436_s_at	890.0	**H2AFX**	H2A histone family, member X
	201476_s_at	904.6	**RRM1**	Ribonucleotide reductase M1 polypeptide
	200910_at	922.9	**CCT3**	Chaperonin containing TCP1, subunit 3 (gamma)
	202330_s_at	924.1	**UNG**	Uracil-DNA glycosylase
	202503_s_at	1060.0	KIAA0101*	KIAA0101
	222077_s_at	1146.2	**RACGAP1**	Rac GTPase activating protein 1
	204170_s_at	1188.5	**CKS2**	CDC28 protein kinase regulatory subunit 2
				
Consensus group 2				
Early	204766_s_at	530.6	**NUDT1**	Nudix (nucleoside diphosphate linked moiety X)-type motif 1
	204825_at	629.0	**MELK**	Maternal embryonic leucine zipper kinase
	203270_at	636.6	**DTYMK**	Deoxythymidylate kinase (thymidylate kinase)
	218585_s_at	768.5	DTL	Denticleless homolog (*Drosophila*)
	220085_at	772.6	**HELLS**	Helicase, lymphoid-specific
Late	205741_s_at	3587.9	DTNA	Dystrobrevin, alpha
	204279_at	3816.5	*PSMB9*	Proteasome (prosome, macropain) subunit, beta type, 9 (large multifunctional peptidase 2)
	204416_x_at	3944.6	APOC1	Apolipoprotein C-I
	202307_s_at	3987.2	*TAP1*	Transporter 1, ATP-binding cassette, sub-family B (MDR/TAP)
	209040_s_at	4243.6	*PSMB8*	Proteasome (prosome, macropain) subunit, beta type, 8 (large multifunctional peptidase 7)
				
Consensus group 3				
Early	204825_at	629.0	**MELK**	Maternal embryonic leucine zipper kinase
	202503_s_at	1060.0	KIAA0101*	KIAA0101
	202705_at	1095.9	**CCNB2**	Cyclin B2
	222077_s_at	1146.2	**RACGAP1**	Rac GTPase activating protein 1
	204170_s_at	1188.5	**CKS2**	CDC28 protein kinase regulatory subunit 2
Late	208997_s_at	3296.1	*UCP2*	Uncoupling protein 2 (mitochondrial, proton carrier)
	205798_at	3499.9	*MMP7*	Matrix metallopeptidase 7 (matrilysin, uterine)
	202307_s_at	3724.7	*PPGB*	Protective protein for beta-galactosidase (galactosialidosis)
	209166_s_at	3946.3	*IL7R*	Interleukin 7 receptor
	206707_x_at	3987.2	*TAP1*	Transporter 1, ATP-binding cassette, sub-family B (MDR/TAP)
	208812_x_at	4485.7	*HLA-C*	Major histocompatibility complex, class I, C

To identify only the most relevant genes, the definition of 'consensus gene set' was tightened from the definition employed in Figure 7. Consensus group 1 = 20 genes overexpressed in at least 15/16 data sets belonging to group 1; consensus group 2 = 58 genes overexpressed in all data sets (6/6) belonging to group 2; consensus group 3 = 29 genes overexpressed in 9/13 data sets belonging to group 3. Bold entries are those expressed more than three times above median in at least one of murine E6, E7, E8, E9, E10 (Symatlas). Italicized entries are those expressed more than three times above median in at least one of the following human cell types: CD4+ T cells, CD8+ T cells, CD19+ B cells (peripheral blood), BDCA4+ dendritic cells, B lymphoblasts (peripheral blood). *Expression data not available in Symatlas.

### A core program of genes expressed in most cancers is active in early development

**Figure 8:**
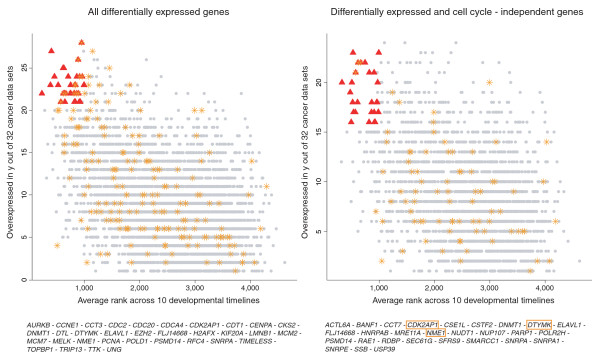
Cancer core program genes before and after cell cycle subtraction. Genes overexpressed in >20/32 data sets and with an average DT rank <1,000 are marked in red and their names are listed below the table (left panel). Analogously for the right panel, with the parameter relaxed to overexpression in >15/32 data sets to account for the reduced number of genes after elimination of CC genes. Genes belonging to the GO category 'cell cycle' are marked as orange asterisks (and with orange boxes in the right panel) to allow a better assessment of the effects of CC subtraction.

To further explore the notion of a tissue-independent core program in cancer, we scored each gene by how many times it is upregulated in all cancer data sets (here, we excluded liver cirrhosis, dysplastic liver and ulcerative colitis) and compared this score to the average rank of the gene across all DTs. Figure 8 shows a highly significant inverse relationship between the developmental rank of a gene and its overexpression frequency (*p *< 2.2e-16). The top-scoring genes in this comparison are related to proliferation and DNA repair and also include transcripts coding for chromatin remodeling proteins (*EZH2*), a histone variant that has recently been linked with stem cell proliferation [28] (*H2AFX*) and RNA-interacting proteins (*ELAVL1*, *SNRPA*). To see whether the relationship between developmental rank and overexpression in cancer is robust towards CC subtraction, we excluded all CC-regulated genes as previously described. The association remains highly significant (*p *< 2.2e-16). Top-scoring genes are mainly involved in RNA processing (*CSTF2*, *SNRPA*, *SNRPA1*, *SNRPE*, *USP39*, *HNRPAB*), which could either be a secondary effect of proliferation or reflect the increased metabolic activity of cancer cells, and chromatin remodeling (*ACTL6A*, *SMARCC1*), indicating that epigenetic mechanisms may be involved in the maintenance of an embryonic phenotype in many cancers.

## Discussion

We have presented a comprehensive, tissue-spanning comparison of gene expression in normal development and human cancer. Main conclusions emerging from this analysis are that a large percentage of tumors recapitulate early developmental gene expression and that the developmental signature in these cancers exhibits low tissue-specificity. Furthermore, we have identified three groups of cancers distinguished by disparate developmental signatures. One group has an early developmental phenotype and expresses genes that are characteristic of stem cells. From a developmental perspective, this group presents very homogeneously. This is all the more surprising as it contains cancer types with complex karyotypes, which are currently thought to lead to more 'chaotic' gene expression. A second, more heterogeneous group tends to be more similar to late development and is characterized by an inflammatory signature. A small group of cancers presents as a transition phenotype between these two extremes and displays both characteristics. This group distinction is reproducible with respect to a new time series of embryonic lung development in the mouse. Finally, we have identified a core program of genes that are expressed in most cancers and mapped the activity of this transcriptional program to early development.

An unexpected result is the low tissue-specificity of the developmental signature, contrasting with previous reports [9,11]. We cannot exclude the possibility that cancer types that were not included in our database recapitulate more tissue-specific developmental patterns. However, our findings suggest that comprehensive comparisons against a diverse set of developmental backgrounds are required before a specific association between a cancer and the development of its cognate tissue can be established on the gene expression level. It is likely that a lineage-specific aspect does exist in cancer gene expression [13], but its magnitude seems to be small in comparison with more generic developmental modules. Possibly, microRNA profiles might be better suited for detection of such subtle signals because they reflect more specific processes than mRNA profiles [29].

Given the type of analysis conducted here, intended to reveal broad brush strokes rather than subtleties, the clear segregation of tumors into three groups with distinct expression patterns is surprising. Clearly, the developmental trajectory provides a meaningful background for capturing large-scale differences in gene expression across diverse conditions. That said, we can only speculate as to what the biological determinants of the observed segregation might be as they are potentially as broad as the contexts in which a proliferative response is 'normal' and physiological.

The capability to divide in response to certain conditions is an inherent property of most cells. Epithelia can augment the production of new cells in response to mechanical irritation [30], fibroblasts divide to reconstitute injured tissue [31], the microvasculature of the female reproductive system periodically expands [32], hepatocytes reconstitute liver tissue after hepatectomy [33] and, of course, cells divide to form a new organism during embryogenesis. The transcriptional programs driving these processes might be as diverse as the contexts that trigger proliferation. Cancers likely exploit endogenous cellular mechanisms to sustain their growth, but our understanding of which of the available paths towards proliferation is chosen in different types of cancers is rudimentary. Our analysis suggests that tumors in group 1 recapitulate an embryonic phenotype: they express early developmental and stem cell genes, suppress genes characteristic of mature tissues and they have downregulated messages required for intercellular communication and signaling. Cell cycle in these tumors might be fueled through the same mechanisms that are employed in rapidly proliferating blastemal cells. Group 3, on the other hand, presents a different picture. Differentially expressed genes imply that proliferation here could occur in the context of wound healing, which is associated with all the processes that are relevant in group 3 (inflammatory reaction, proliferation, tissue remodeling). The conception of cancer as a 'wound that does not heal' has often been cited [34]. Our analysis suggests that it might be more applicable to some tumors than to others. Indeed, the clinical behavior of tumors in group 3 seems to exhibit some special features. Even though ovarian cancer is known as a malignancy with poor prognosis, its growth rate often is slow and patients can live with metastases for years [14]. Renal cell carcinoma also has a poor prognosis when metastatic; however, most renal cell carcinomas have an indolent growth rate [15]. Finally, thyroid cancers are also recognized for their slow growth [17]. It is not clear whether the inflammatory gene expression signature we observe in these tumors is a cause or consequence of this particular behavior, but further investigation of this question has profound clinical implications. If tumors truly rely on distinct programs for proliferation and survival, a classification system that takes these differences into consideration could provide valuable guidelines for therapeutic decisions. Based on our study, for example, we would predict that a drug interfering with the wound healing program might be effective against both ovarian and renal carcinoma, but not against Wilms' tumor or lung adenocarcinoma. Interestingly, a recent paper that examined gene expression in mouse models of colon carcinoma in a developmental context revealed a distinction between *Smad3-/- *and *Tgfb1-/-; Rag2-/- *models (both exhibiting a strong inflammatory component and showing similarity to late colon development) and *ApcMin/+ *and *AOM *models, which recapitulated early colon development [10]. This result implies that different genetic alterations might underlie the distinct gene expression signatures in group 1 and 3 cancers.

To refine the distinction between the developmental groups of cancers emerging from our analysis, more data - ideally acquired under standardized conditions - are necessary. While the embryonic cancers in group 1 seem to represent a fairly homogeneous population with respect to their developmental component, diseases in group 3 are far more heterogeneous and more reliable data would probably lead to further sub-classification of these cancers. Standardized data would also likely help to resolve the group affiliation of ambiguous cases like thyroid carcinoma. Both PTC data sets mapping to group 3 are paired experiments, with tumor and normal tissue coming from the same patient, while the PTC data set in group 1 is unpaired. Such differences seem to have a larger impact on the genes that are identified as differentially expressed than commonly assumed. A recent study elegantly proves this point by showing an altered gene expression signature in 'normal' tissue adjacent to lung tumors [35]. Other possibly confounding factors like the degree of lymphocyte infiltration in different samples and the already mentioned specification of histological subtype might play important roles in determining the developmental profile of a tumor and should be accounted for in future studies.

To gain a better understanding of the biology underlying different loci on the developmental landscape, it might also be helpful to include more pathological conditions unrelated to cancer in the analysis. For many diseases, we have sufficiently good understanding of etiology and pathophysiology to be able to use them as 'landing lights' on the developmental surface.

## Conclusion

The results presented here suggest that there is great potential for better understanding of human disease in a 'macrobiological' approach to analyzing high-throughput data. Shifting our focus from single sets of genes or processes to the biology of aggregates on the order of the entire transcriptome is likely to be useful in establishing highly robust molecular correlations between seemingly unrelated disease phenotypes.

## Materials and methods

### Data

All gene expression data with the exception of the lung development validation series came from the public domain. Developmental time courses were profiled on several different Affymetrix chips (MG-430 2.0, MG-430A, MG-U74, Mu11K, HG-U133A). To exclude potential platform-related bias, we restricted ourselves to Affymetrix HG-U133A or HG-U133 Plus 2.0 arrays for cancer gene expression profiles. A detailed description of all data sets can be found in Additional data file 6.

### Data preprocessing

When available, .CEL files were downloaded and arrays were normalized and expression measures calculated using the robust multi-array average [36,37]. When raw data were not available, MAS5 preprocessed expression values were downloaded, quantile-normalized and log2-transformed.

### Cross-platform comparison and homology mapping

On Affymetrix arrays, a gene is often assayed by several probe sets. We first reduced each platform to unique Entrez Gene IDs. To avoid artifacts in downstream analyses caused by biased probe set selection, we randomly chose the probe set that would represent a gene on a particular platform. Probe sets with no Entrez ID were removed. In the next step, we used the homologene database (NCBI) to define orthologs between the human and the mouse. Entrez IDs with no ortholog were removed from all platforms. Finally, we matched orthologous genes across platforms. The resulting 'consensus' between the different platforms consists of 5,166 unique genes.

### Construction of the developmental timeline: principal components analysis

We used principal components analysis to construct a DT for each developmental time series [11]. Principal components analysis is a linear data transformation technique that allows representation of the original data in a new coordinate system in which the axes (principal components (PCs)) are chosen to capture as much variation in the data as possible in a decreasing order, that is, PC1 accounts for x% variability, PC2 for y%, PC3 for z% and so on, with x > y > z. We first normalized the expression values of each gene across conditions (time points) to mean 0 and standard deviation 1. Principal components analysis was carried out on the normalized data using the R language and environment for statistical computing [38], with genes representing objects and time points representing the features whose dimensionality is to be reduced. For the purposes of our analysis, we were interested in the PC that is most significantly associated with time. For each developmental time series M (5,166 rows/genes and *k* columns/time points), we therefore computed the correlation between a time vector (1,2,3...,*k*) and the component loadings for each of the *k *PCs. For all time series with the exception of liver regeneration, PC1 was most significantly correlated with the time vector (>0.8). For liver regeneration, the highest correlation (~0.6) was found for PC3, indicating that the major changes in gene expression during liver development do not occur in a continuum from time point 1 to *k*, as in development. The most active stage of hepatocyte regeneration occurs approximately 48 h after hepatectomy, while our time series spans 0-72 h (Seth Karp, personal communication).

The DT of a developmental time series is the original data matrix M (5,166 rows/genes and *k *columns/time points) after the transformation:

*y *= *v*^*T *^× *d*^*T*^

where *v*^*T *^denotes the *k*-dimensional PC of M that is most significantly correlated with time.

### Analysis of differential gene expression and construction of developmental profiles

Differential gene expression between tumors and corresponding controls was determined using significance analysis of microarrays (SAM) [39]. All genes with a q-value <0.05 were considered differentially expressed. For purposes of consistency, SAM based on an unpaired *t*-test was used for all data sets, even though paired data were available in four cases.

### Frequency plots and probability distributions

Frequency plots were constructed by dividing the DTs in 13 equally sized (approximately 400 genes) compartments and plotting the compartment index against the number of upregulated and downregulated genes mapping to that compartment.

Probability distributions show the probability *P*(*DEV*[1,2,...*i*]|*cancer*) of being among the first *i* genes on the DT (positions are numbered 1-5,166, starting on the left, early side) if deregulated in cancer for up- and downregulated genes. Specifically, we plot:

$$P\left(DEV\left[1,2,...i\right]|cancer\right)=\frac{n\left(deregulated\cap DEV\left[1,2,...i\right]\right)}{n\left(deregulated\right)}\text{ for }i=2,3,4...5,166.$$

We then quantified the shape of the distribution by fitting two independent linear models to the data, one regression line representing the probability distribution on the early end of the DT and another one approximating the distribution on the late end (illustrated in Figure 1b and top right corner of Figure 3). The goal was to find two regression lines that best approximate the probability distribution and use their slopes as a two-dimensional summary of how the cancer genes map to the DT. Since each probability distribution has a unique shape, it has to be determined in each individual case at which point on the DT the breakpoint between the early and late model should occur to achieve an optimal approximation. We computed a series of F statistics (Chow test) for each potential breakpoint in the probability distribution; that is, we tested how different the coefficients of the two regression lines are if we choose the breakpoint at *DEV*[*i*] for *i *= 774,775,...4,391 (this excludes the earliest and latest parts of the DT because the linear model should represent a sufficiently large segment). The optimal breakpoint is defined as the maximal value in the series of F statistics. All computations were done using the strucchange package available at [40]. The fit of the regression lines to all probability distributions (blue lines) can be viewed in Additional data files 9 (before cell cycle subtraction) and 10 (after cell cycle subtraction). For each combination of cancer and DT, this approach yields four regression lines: two models representing early and late probabilities for upregulated genes and two models for downregulated genes. For each cancer, we can thus summarize the relationship to the 10 DTs in a 40-dimensional vector (4 regression line slopes × 10 DTs). These vectors were for used for clustering using Euclidian distance and Ward's linkage (Figure 3).

### Cell cycle subtraction

We downloaded cell cycle scores for 38,578 probes [41]. The scaled Fourier periodicity scores ranged from 0.1-58; the cutoff for being considered cell cycle regulated in the original publication was 3.2. In our CC subtracted data sets, we allowed only genes with a defined score <1.

### Meta-developmental signatures, consensus gene sets and GO characterization

The meta-developmental signatures were determined by computing the average rank of each gene across all ten DTs and selecting the x genes with earliest (eDEVx) and latest (lDEVx) expression. Enrichment of GO categories in meta-developmental signatures and deregulated genes in cancer was determined against the background of all 5,166 genes in our analysis using Bioconductor's GOstats package [42]. Consensus gene sets for tumors in groups 1-3 were defined as those genes that are upregulated (downregulated) in at least 60% of datasets belonging to a given group, that is, 11/15 for group 1, 5/6 for group 2 and 8/13 for group 3.

### C2 gene set enrichment

We downloaded all C2 gene sets from the Broad Institute website [43], eliminated the fraction that had an overlap of less than 15 genes with our data sets, and augmented the C2 collection with 10 meta-developmental gene signatures (DEV30, 50, 100, 200, 500 and LATEDEV30, 50, 100, 200, 500). We then tested the enrichment of each of these 999 gene signatures in the up- and downregulated genes of our 32 data sets using Fisher's exact test (one-sided). Clustering of all data sets by the *p*-values for the top 20 enriched gene sets in groups 1-3 was accomplished using Manhattan distance and Ward's linkage.

R scripts for all above-mentioned analyses are provided on the website accompanying this paper [44].

### Validation time series: embryonic lung development

Whole lungs were dissected from C57BL/6J mice at E11.5, E13.5, E14.5, E16.5 and postnatal day 5 and stored in RNAlater (Ambion, Austin, TX, USA). All time points represent gene expression patterns of individuals; only E11.5 was a pooled sample (seven pups). Total RNA was extracted using Ambion's mirVana miRNA isolation kit and tested for quality using a bioanalyzer (Agilent, Santa Clara, CA, USA). RNA integrity numbers ranged from 9.2-9.7. The samples were prepared for hybridization to Affymetrix Mouse 430 2.0 arrays according to the manufacturer's instructions. Processed and raw data have been submitted to Gene Expression Omnibus [45] under accession number GSE11539 and are also available in RMA-normalized form as Additional data file 7.

## Abbreviations

CC, cell cycle; CRCC, clear cell renal cell carcinoma; DT, developmental timeline; E, embryonic day; ES, embryonic stem; ESEN, early secretory endometrium; GO, Gene Ontology; PC, principal component; PEN, proliferative endometrium; PTC, papillary thyroid carcinoma; SAM, significance analysis of microarrays.

## Authors' contributions

ISK, SK and KN conceived of and designed the study. KN carried out all analyses and wrote the manuscript. ISK, SK, CJB, AP and BBK provided guidance and participated in the preparation of the manuscript. CJB, AP, KF and KN performed the experimental work.

## Additional data files

The following additional data are available. Additional data file 1 contains the frequency plots for all cancer types and all developmental time series. Additional data file 2 contains the frequency plots for all cancers and all time series after CC subtraction. Additional data file 3 shows the frequency plots for the top 450 differentially expressed genes in CRCC1 and CRCC2. Additional data file 4 contains a heatmap of probability distribution slopes after CC subtraction (analogously to Figure 3). Additional data file 5 is a heatmap of enrichment *p*-values for gene sets that ranked among the 20 most enriched in the downregulated genes of either group 1, 2 or 3. Additional data file 6 is a spreadsheet containing detailed annotation for all data sets used in this study. Additional data file 7 contains the raw data for the lung development validation time series (after RMA-normalization). Additional data file 8 contains a smooth histogram of early upregulated (UpE) probability distribution slopes for all cancer data sets. Additional data file 9 contains the probability distribution plots and linear regression fits for all cancers and all time series. Additional data file 10 shows the same data as additional data file 9, but after CC subtraction.

## Supplementary Material

Additional data file 1 Frequency plots for all cancer types and all developmental time series. Click here for file

Additional data file 2 Frequency plots for all cancers and all time series after CC subtraction. Click here for file

Additional data file 3 Frequency plots for the top 450 differentially expressed genes in CRCC1 and CRCC2. Click here for file

Additional data file 4 Heatmap of probability distribution slopes after CC subtraction (analogously to Figure 3). Click here for file

Additional data file 5 Heatmap of enrichment *p*-values for gene sets that ranked among the 20 most enriched in the downregulated genes of either group 1, 2 or 3. Click here for file

Additional data file 6 Detailed annotation for all data sets used in this study. Click here for file

Additional data file 7 Raw data for the lung development validation time series (after RMA-normalization). Click here for file

Additional data file 8 This shows a bimodal distribution with the left peak containing group 3 tumors, the right peak containing group 1 tumors and intermediate cases (group 2) falling in between. Click here for file

Additional data file 9 Probability distribution plots and linear regression fits for all cancers and all time series. Click here for file

Additional data file 10 The same data as additional data file 9, but after CC subtraction. Click here for file
