# Supplementary material for: Analysis of gene expression in a developmental context emphasizes distinct biological leitmotifs in human cancers
Source: Genome Biol. 2008 Jul 8;9(7):R108. doi: 10.1186/gb-2008-9-7-r108 (PMC2530866; doi:10.1186/gb-2008-9-7-r108)
Supplement: Additional data file 1 — Frequency plots for all cancer types and all developmental time series. [file gb-2008-9-7-r108-S1.pdf]

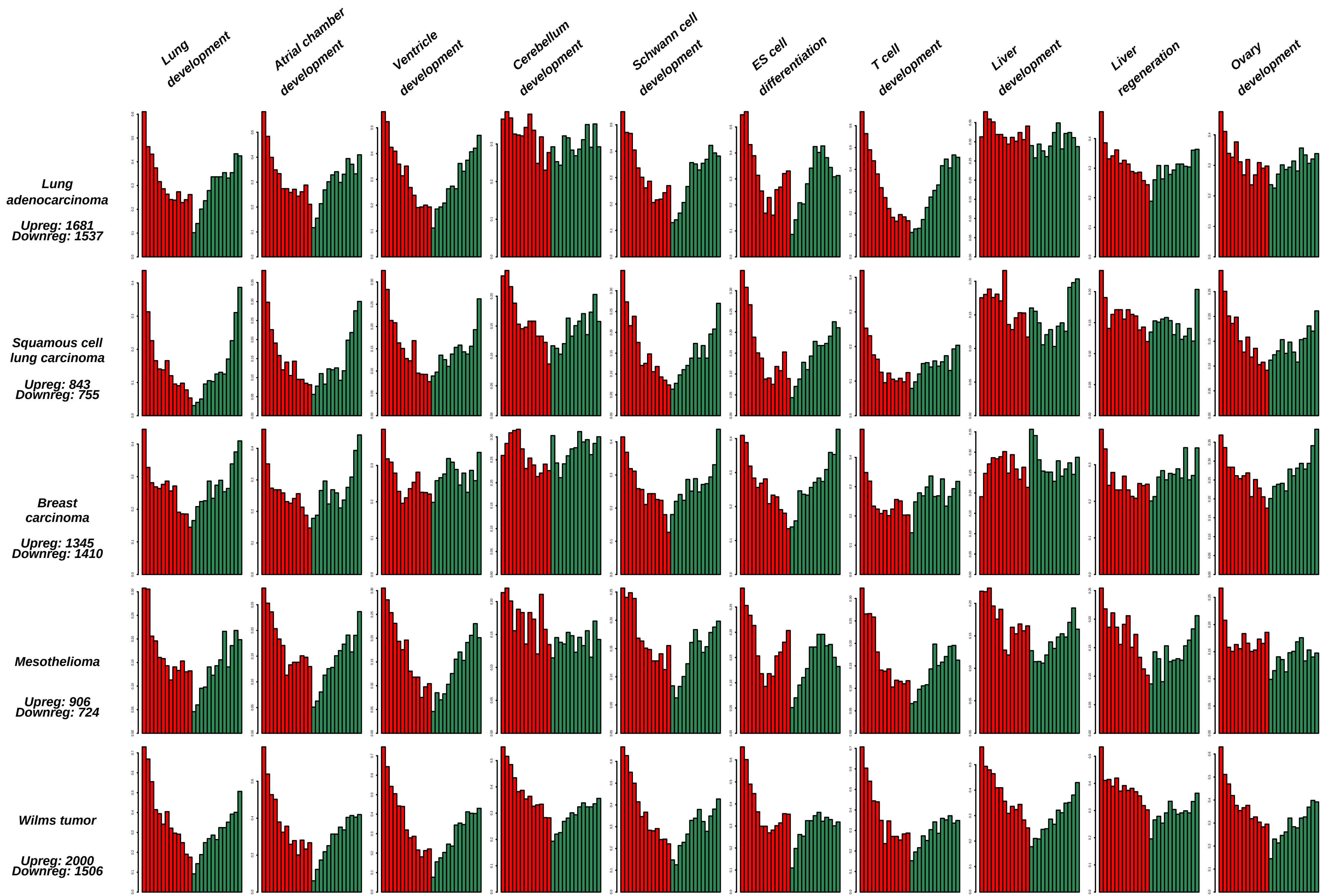
Lung development
Atrial chamber development
Ventricle development
Cerebellum development
Schwann cell development
ES cell differentiation
T cell development
Liver development
Liver regeneration
Ovary development
Lung adenocarcinoma
Upreg: 1681
Downreg: 1537
Squamous cell lung carcinoma
Upreg: 843
Downreg: 755
Breast carcinoma
Upreg: 1345
Downreg: 1410
Mesothelioma
Upreg: 906
Downreg: 724
Wilms tumor
Upreg: 2000
Downreg: 1506

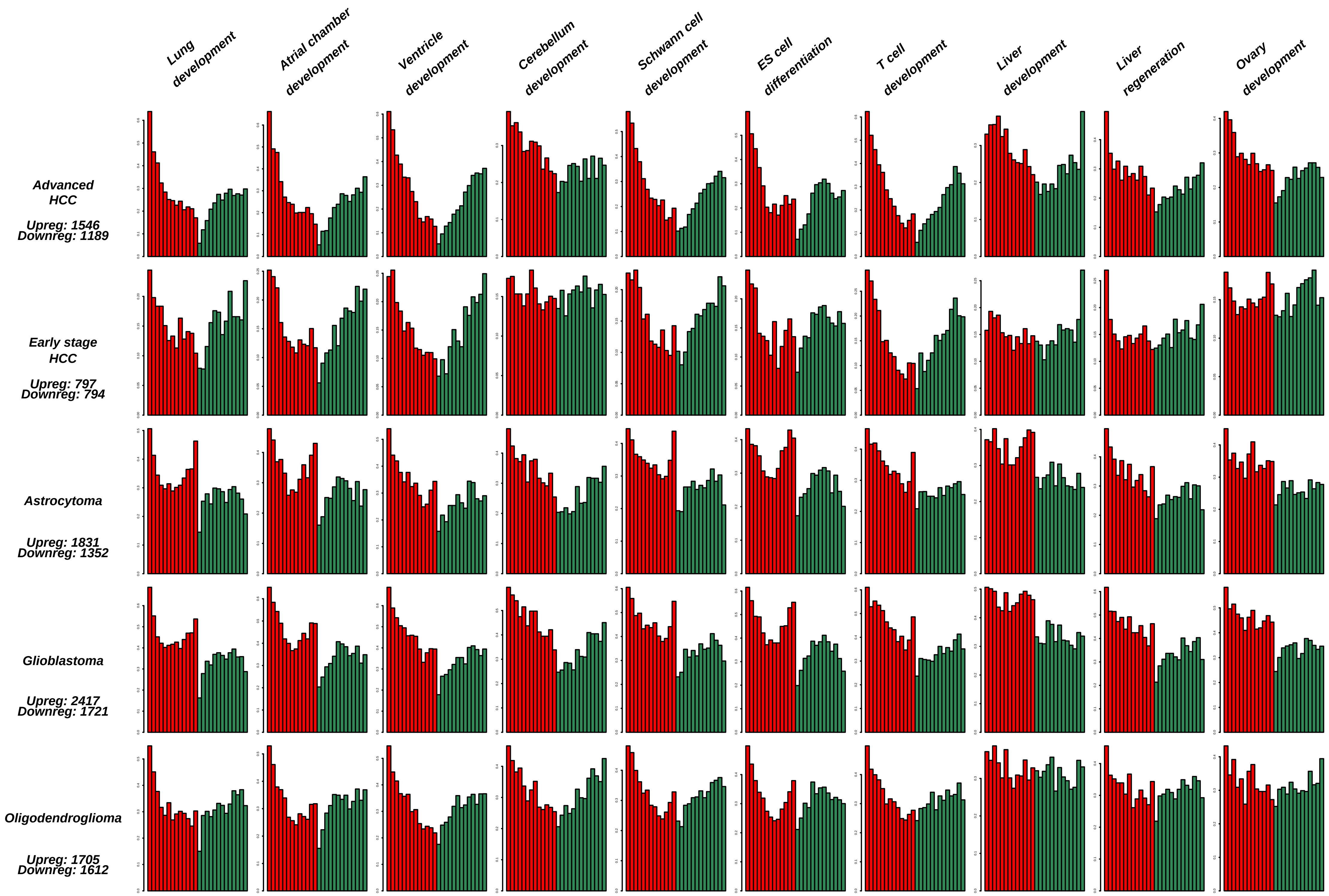

Lung development
Atrial chamber development
Ventricle development
Cerebellum development
Schwann cell development
ES cell differentiation
T cell development
Liver development
Liver regeneration
Ovary development
Advanced HCC
Upreg: 1546
Downreg: 1189
Early stage HCC
Upreg: 797
Downreg: 794
Astrocytoma
Upreg: 1831
Downreg: 1352
Glioblastoma
Upreg: 2417
Downreg: 1721
Oligodendroglioma
Upreg: 1705
Downreg: 1612

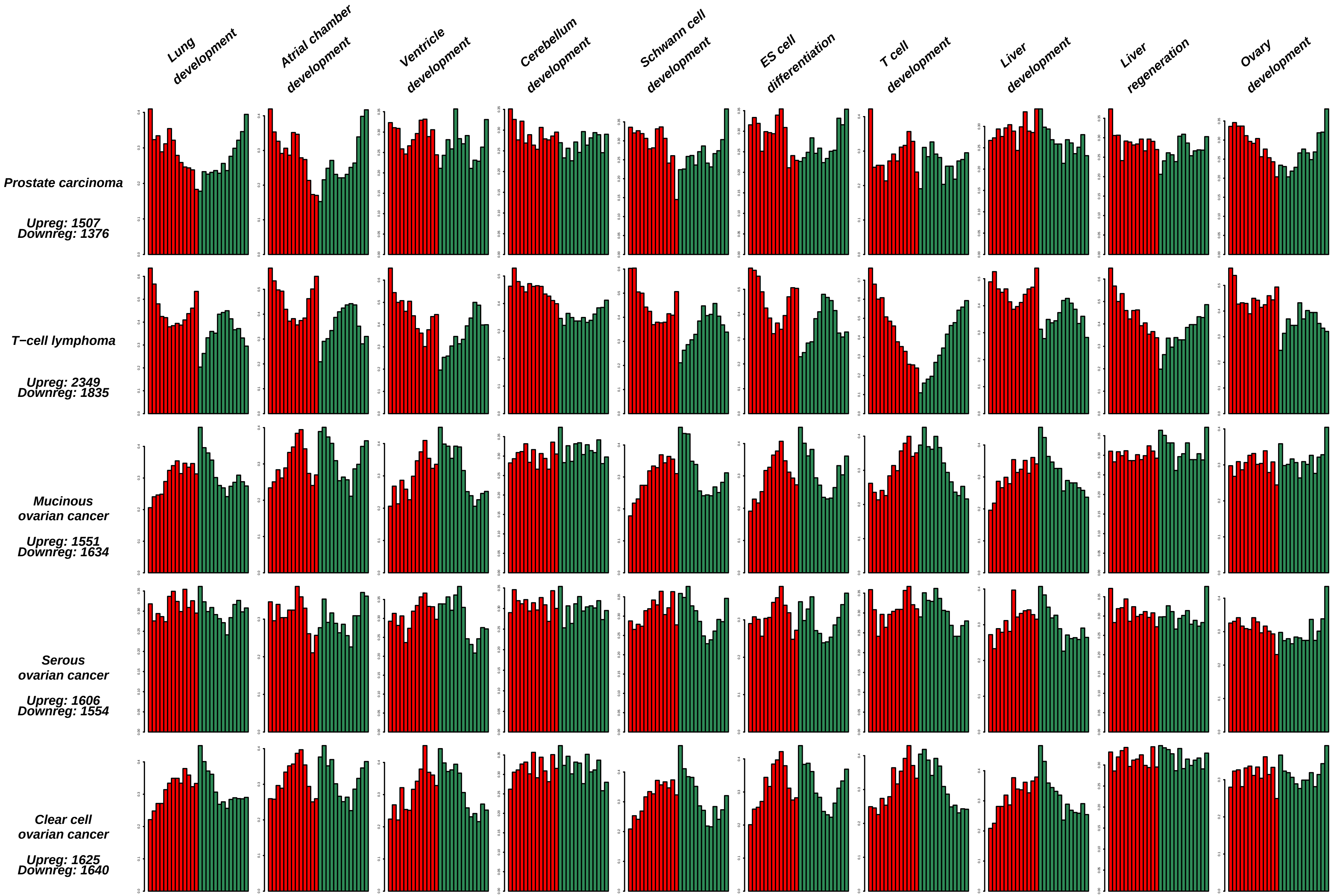
Lung development
Atrial chamber development
Ventricle development
Cerebellum development
Schwann cell development
ES cell differentiation
T cell development
Liver development
Liver regeneration
Ovary development
Prostate carcinoma
Upreg: 1507
Downreg: 1376
T−cell lymphoma
Upreg: 2349
Downreg: 1835
Mucinous ovarian cancer
Upreg: 1551
Downreg: 1634
Serous ovarian cancer
Upreg: 1606
Downreg: 1554
Clear cell ovarian cancer
Upreg: 1625
Downreg: 1640

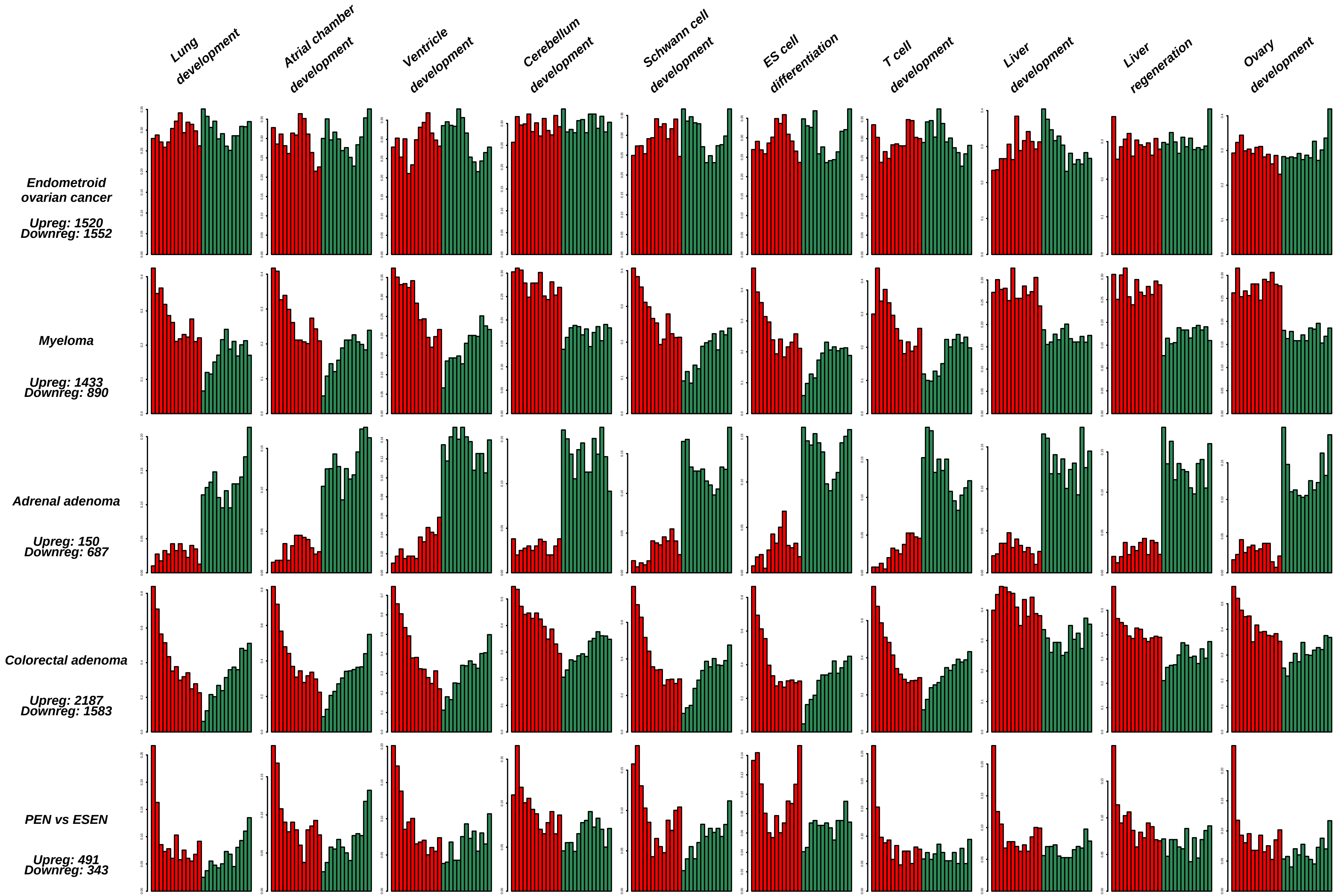
Lung development
Atrial chamber development
Ventricle development
Cerebellum development
Schwann cell development
ES cell differentiation
T cell development
Liver development
Liver regeneration
Ovary development
Endometroid ovarian cancer
Upreg: 1520
Downreg: 1552
Myeloma
Upreg: 1433
Downreg: 890
Adrenal adenoma
Upreg: 150
Downreg: 687
Colorectal adenoma
Upreg: 2187
Downreg: 1583
PEN vs ESEN
Upreg: 491
Downreg: 343

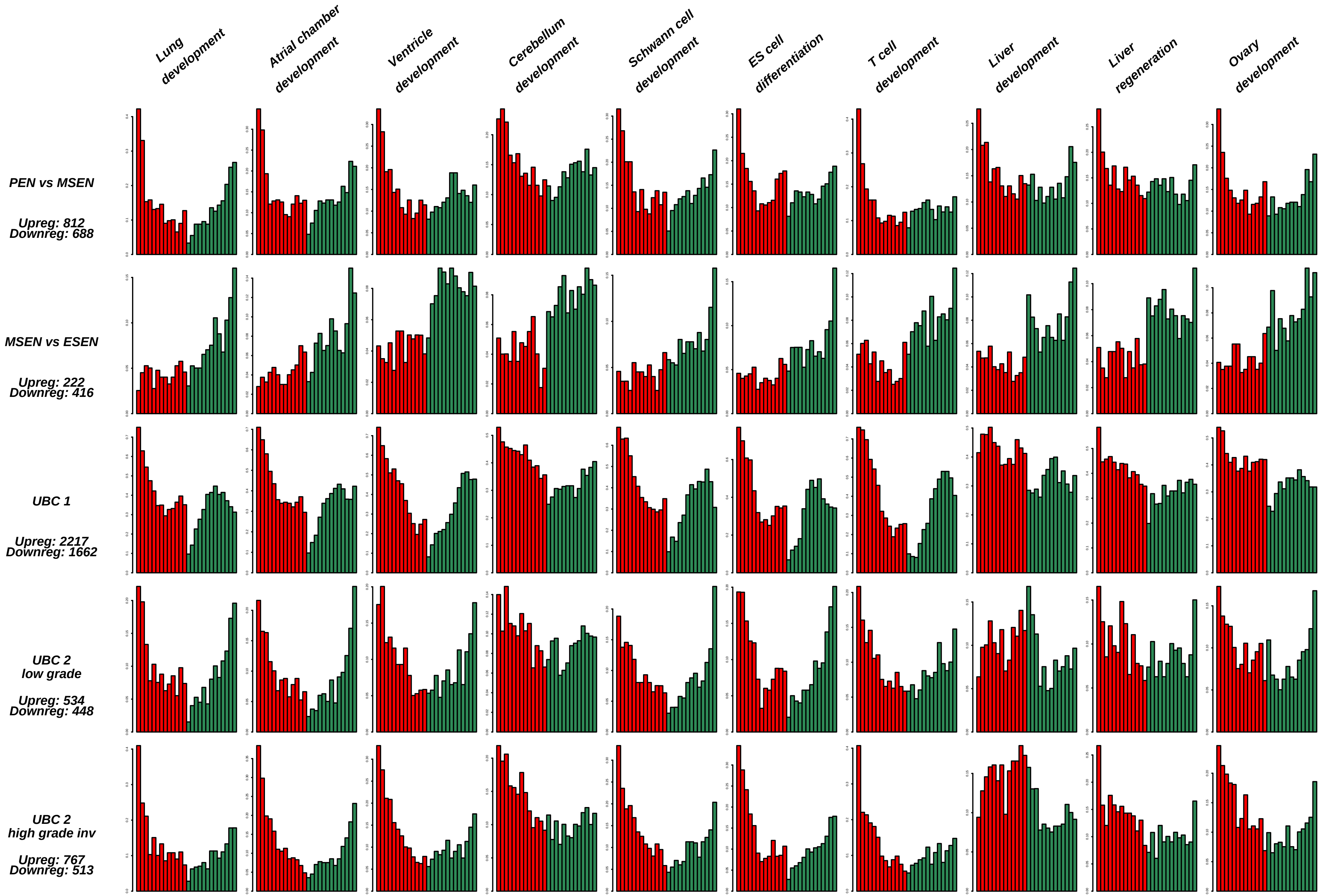
Lung development
Atrial chamber development
Ventricle development
Cerebellum development
Schwann cell development
ES cell differentiation
T cell development
Liver development
Liver regeneration
Ovary development
PEN vs MSEN
Upreg: 812
Downreg: 688
MSEN vs ESEN
Upreg: 222
Downreg: 416
UBC 1
Upreg: 2217
Downreg: 1662
UBC 2
low grade
Upreg: 534
Downreg: 448
UBC 2
high grade inv
Upreg: 767
Downreg: 513

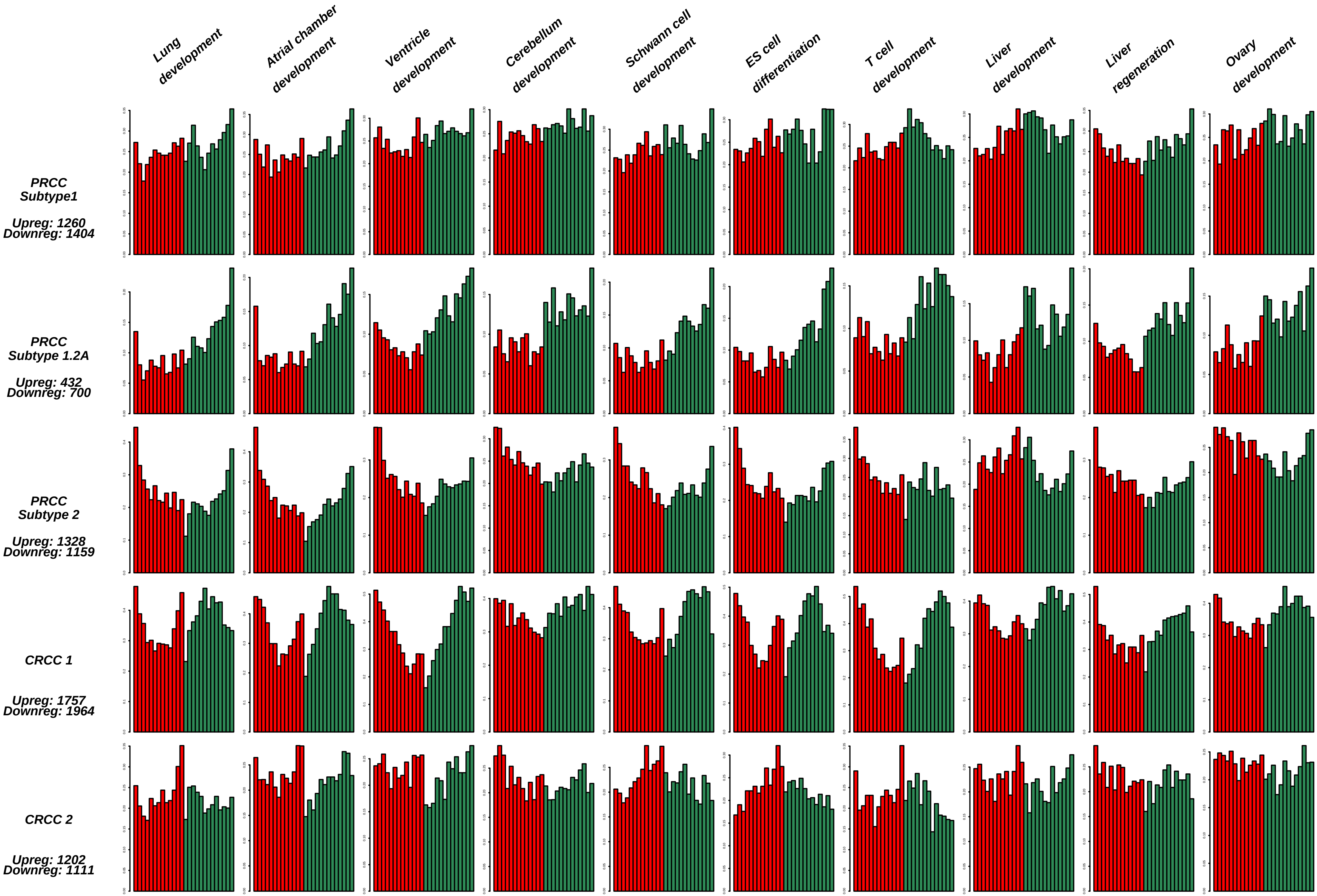
Lung development
Atrial chamber development
Ventricle development
Cerebellum development
Schwann cell development
ES cell differentiation
T cell development
Liver development
Liver regeneration
Ovary development
PRCC Subtype1
Upreg: 1260
Downreg: 1404
PRCC Subtype 1.2A
Upreg: 432
Downreg: 700
PRCC Subtype 2
Upreg: 1328
Downreg: 1159
CRCC 1
Upreg: 1757
Downreg: 1964
CRCC 2
Upreg: 1202
Downreg: 1111

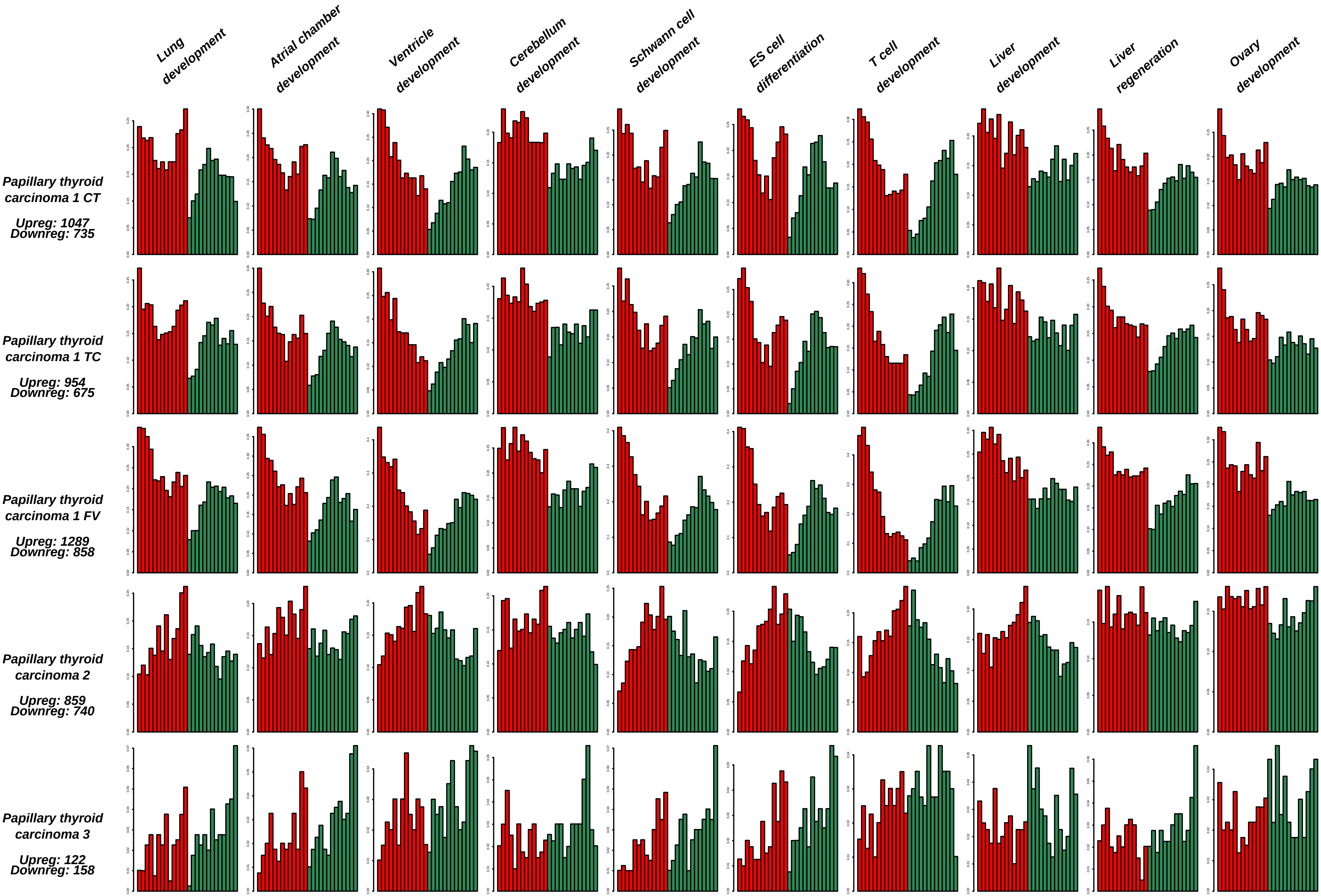

Lung development
Atrial chamber development
Ventricle development
Cerebellum development
Schwann cell development
ES cell differentiation
T cell development
Liver development
Liver regeneration
Ovary development
Papillary thyroid carcinoma 1 CT
Upreg: 1047
Downreg: 735
Papillary thyroid carcinoma 1 TC
Upreg: 954
Downreg: 675
Papillary thyroid carcinoma 1 FV
Upreg: 1289
Downreg: 858
Papillary thyroid carcinoma 2
Upreg: 859
Downreg: 740
Papillary thyroid carcinoma 3
Upreg: 122
Downreg: 158

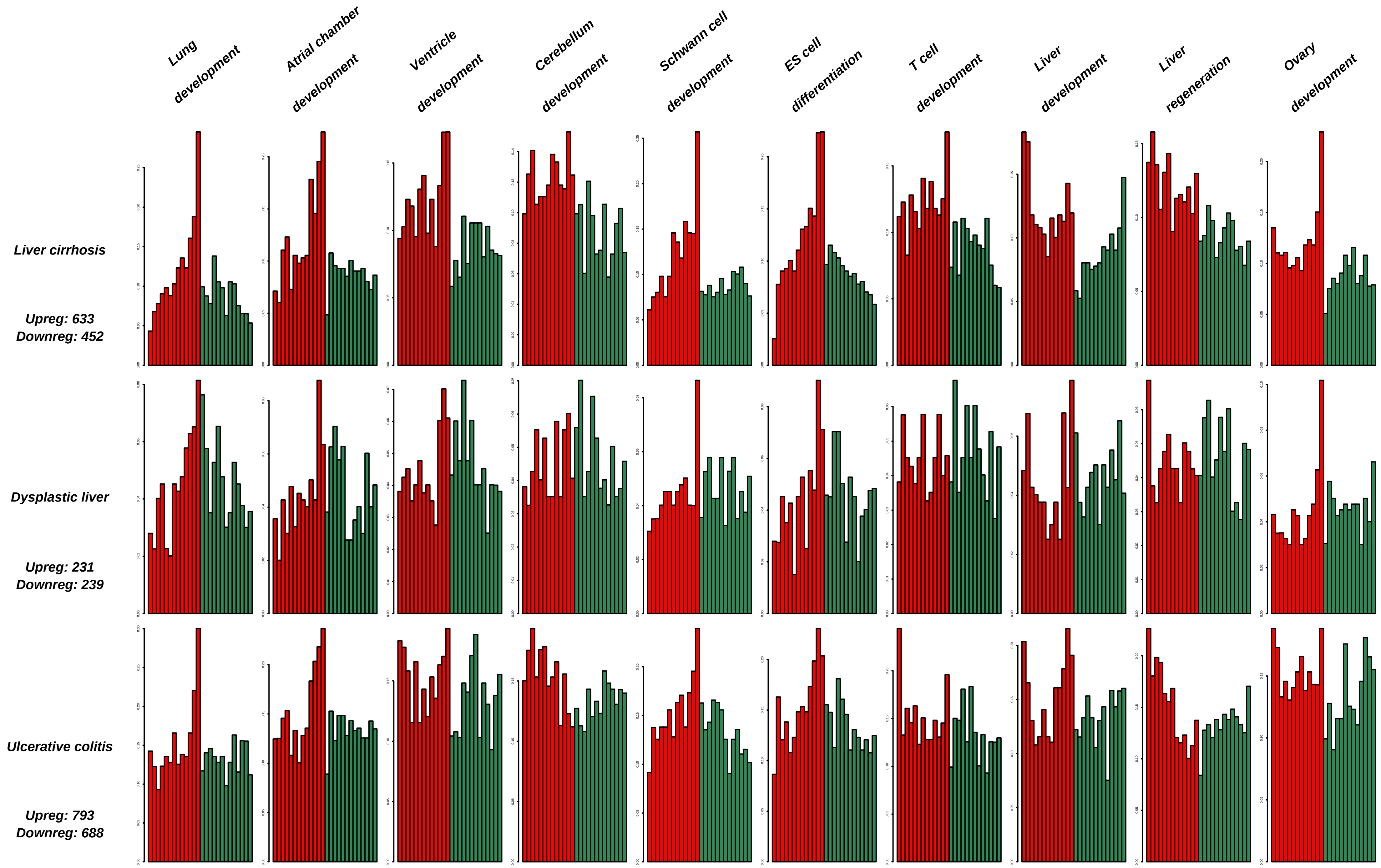

Lung development
Atrial chamber development
Ventricle development
Cerebellum development
Schwann cell development
ES cell differentiation
T cell development
Liver development
Liver regeneration
Ovary development
Liver cirrhosis
Upreg: 633
Downreg: 452
Dysplastic liver
Upreg: 231
Downreg: 239
Ulcerative colitis
Upreg: 793
Downreg: 688
