# Supplementary material for: Analysis of gene expression in a developmental context emphasizes distinct biological leitmotifs in human cancers
Source: Genome Biol. 2008 Jul 8;9(7):R108. doi: 10.1186/gb-2008-9-7-r108 (PMC2530866; doi:10.1186/gb-2008-9-7-r108)
Supplement: Additional data file 2 — Frequency plots for all cancers and all time series after CC subtraction. [file gb-2008-9-7-r108-S2.pdf]

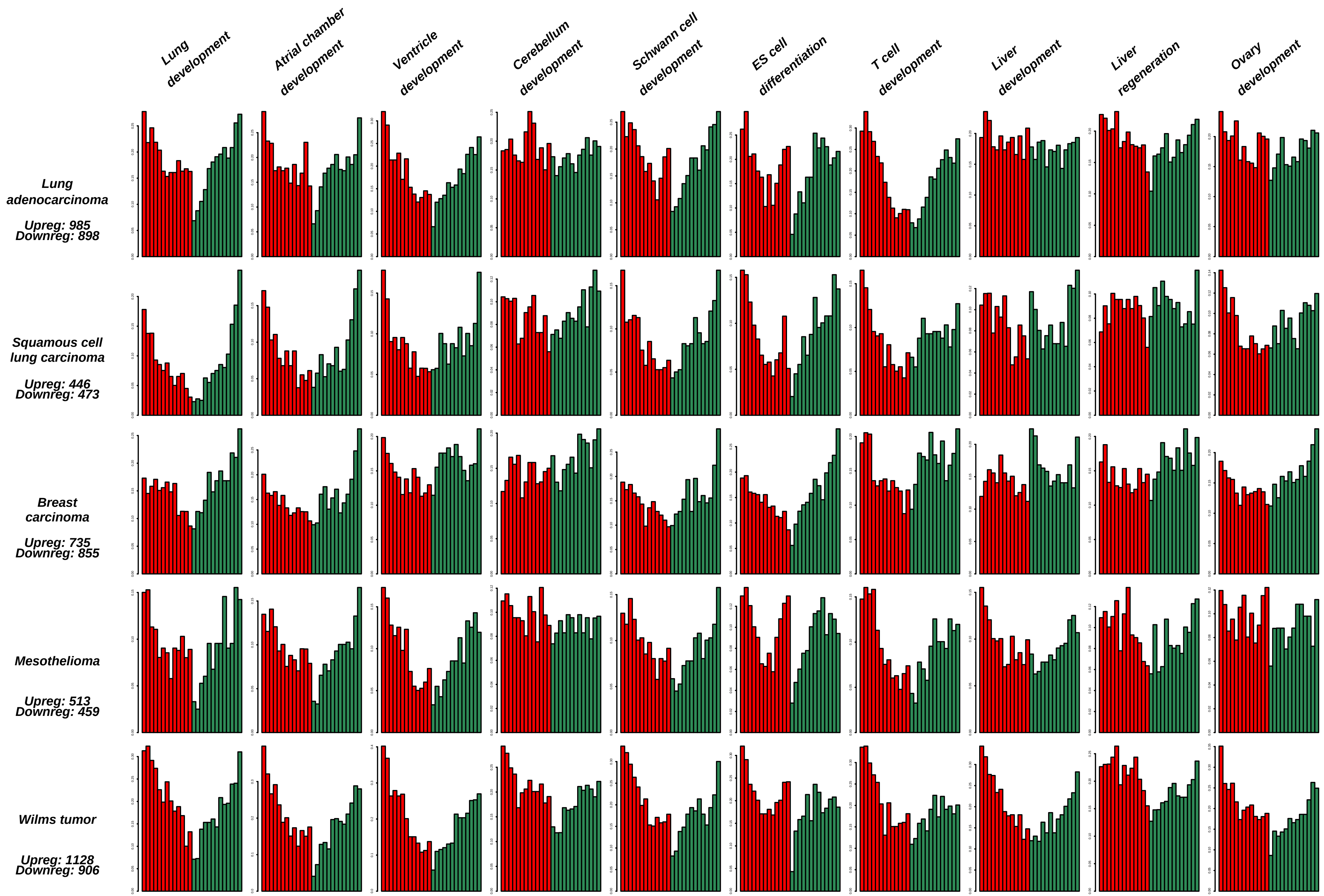

Lung development
Atrial chamber development
Ventricle development
Cerebellum development
Schwann cell development
ES cell differentiation
T cell development
Liver development
Liver regeneration
Ovary development
Lung adenocarcinoma
Upreg: 985
Downreg: 898
Squamous cell lung carcinoma
Upreg: 446
Downreg: 473
Breast carcinoma
Upreg: 735
Downreg: 855
Mesothelioma
Upreg: 513
Downreg: 459
Wilms tumor
Upreg: 1128
Downreg: 906

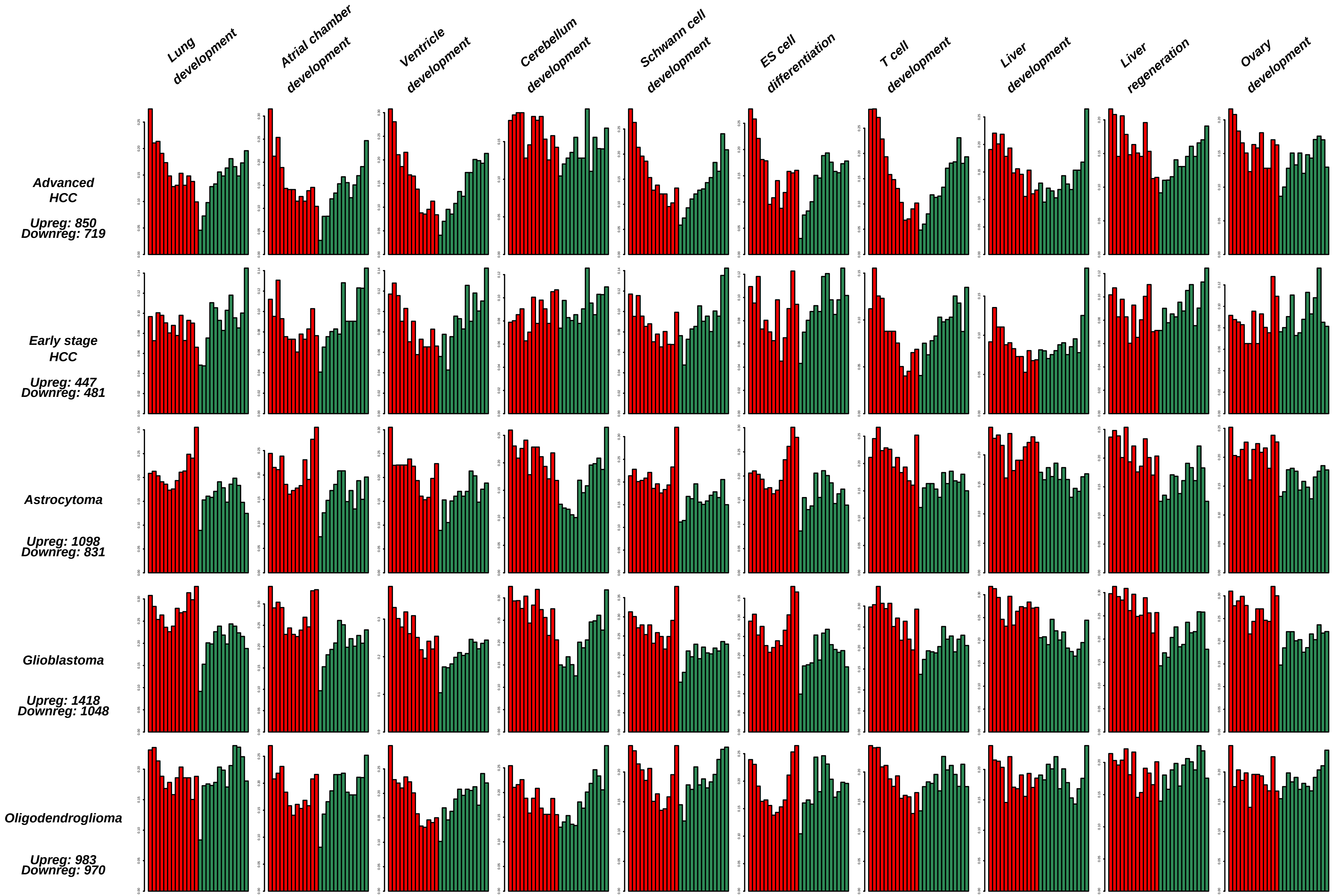
Lung development
Atrial chamber development
Ventricle development
Cerebellum development
Schwann cell development
ES cell differentiation
T cell development
Liver development
Liver regeneration
Ovary development
Advanced HCC
Upreg: 850
Downreg: 719
Early stage HCC
Upreg: 447
Downreg: 481
Astrocytoma
Upreg: 1098
Downreg: 831
Glioblastoma
Upreg: 1418
Downreg: 1048
Oligodendroglioma
Upreg: 983
Downreg: 970

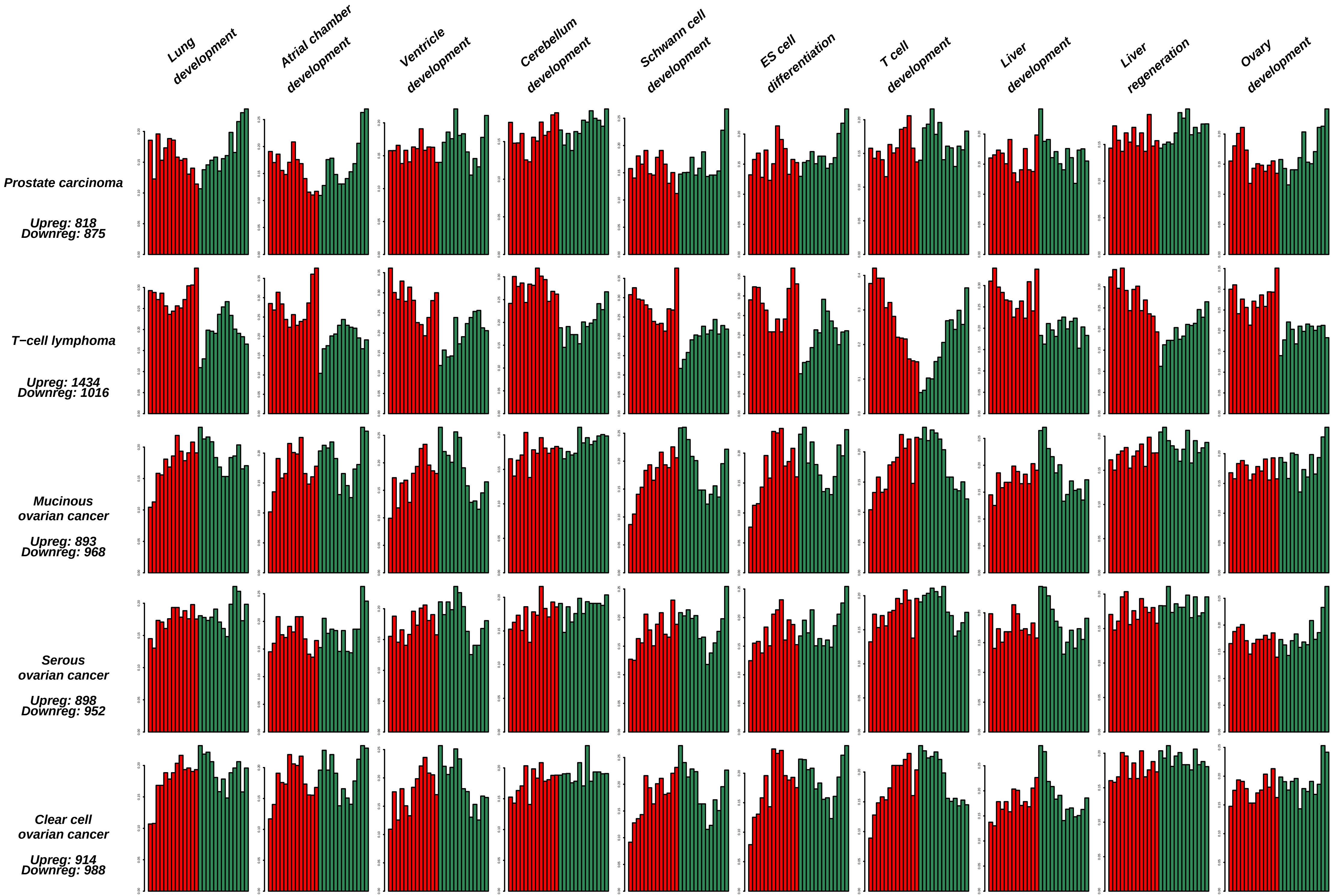
Lung development
Atrial chamber development
Ventricle development
Cerebellum development
Schwann cell development
ES cell differentiation
T cell development
Liver development
Liver regeneration
Ovary development
Prostate carcinoma
Upreg: 818
Downreg: 875
T−cell lymphoma
Upreg: 1434
Downreg: 1016
Mucinous ovarian cancer
Upreg: 893
Downreg: 968
Serous ovarian cancer
Upreg: 898
Downreg: 952
Clear cell ovarian cancer
Upreg: 914
Downreg: 988

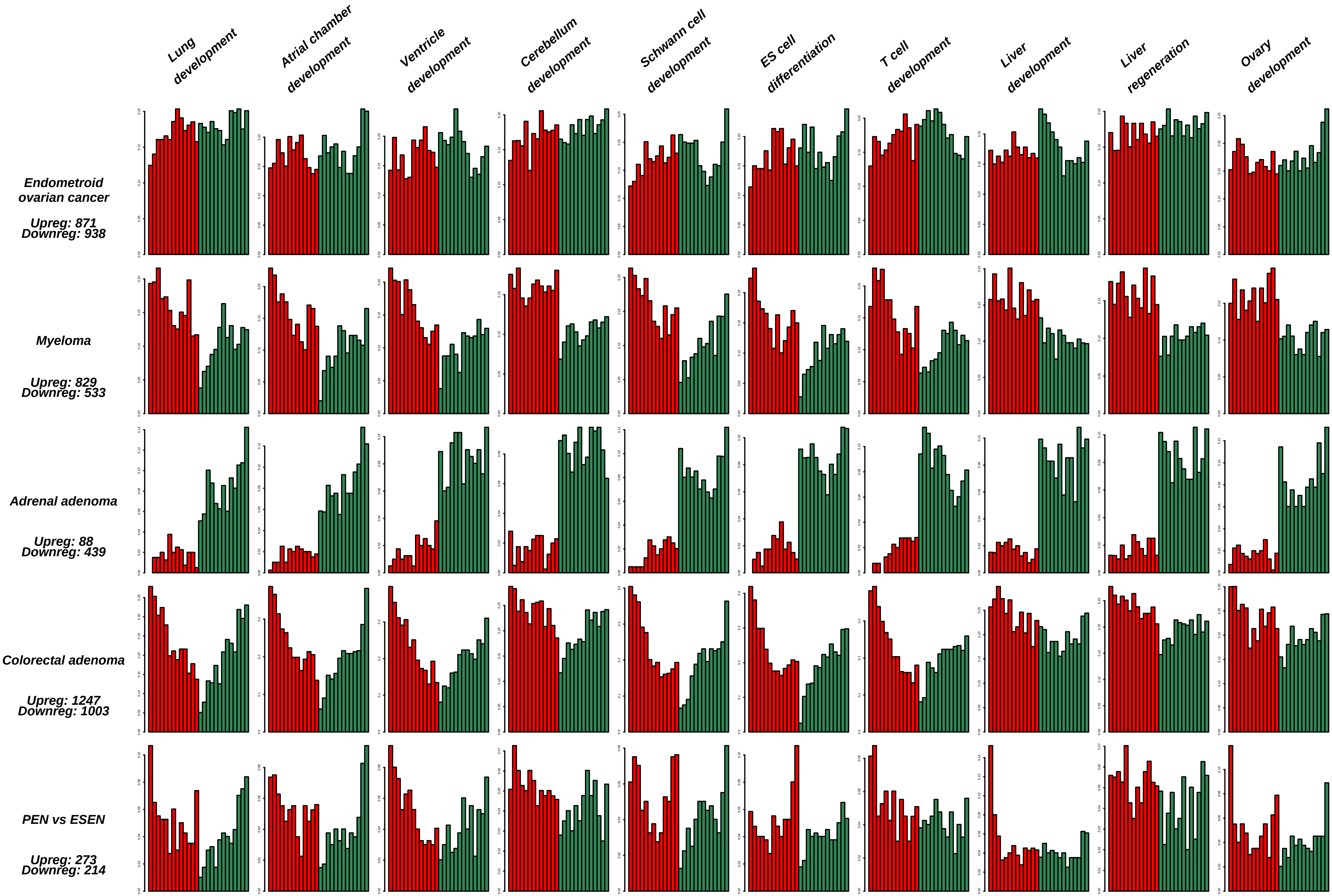

Lung development
Atrial chamber development
Ventricle development
Cerebellum development
Schwann cell development
ES cell differentiation
T cell development
Liver development
Liver regeneration
Ovary development
Endometroid ovarian cancer
Upreg: 871
Downreg: 938
Myeloma
Upreg: 829
Downreg: 533
Adrenal adenoma
Upreg: 88
Downreg: 439
Colorectal adenoma
Upreg: 1247
Downreg: 1003
PEN vs ESEN
Upreg: 273
Downreg: 214

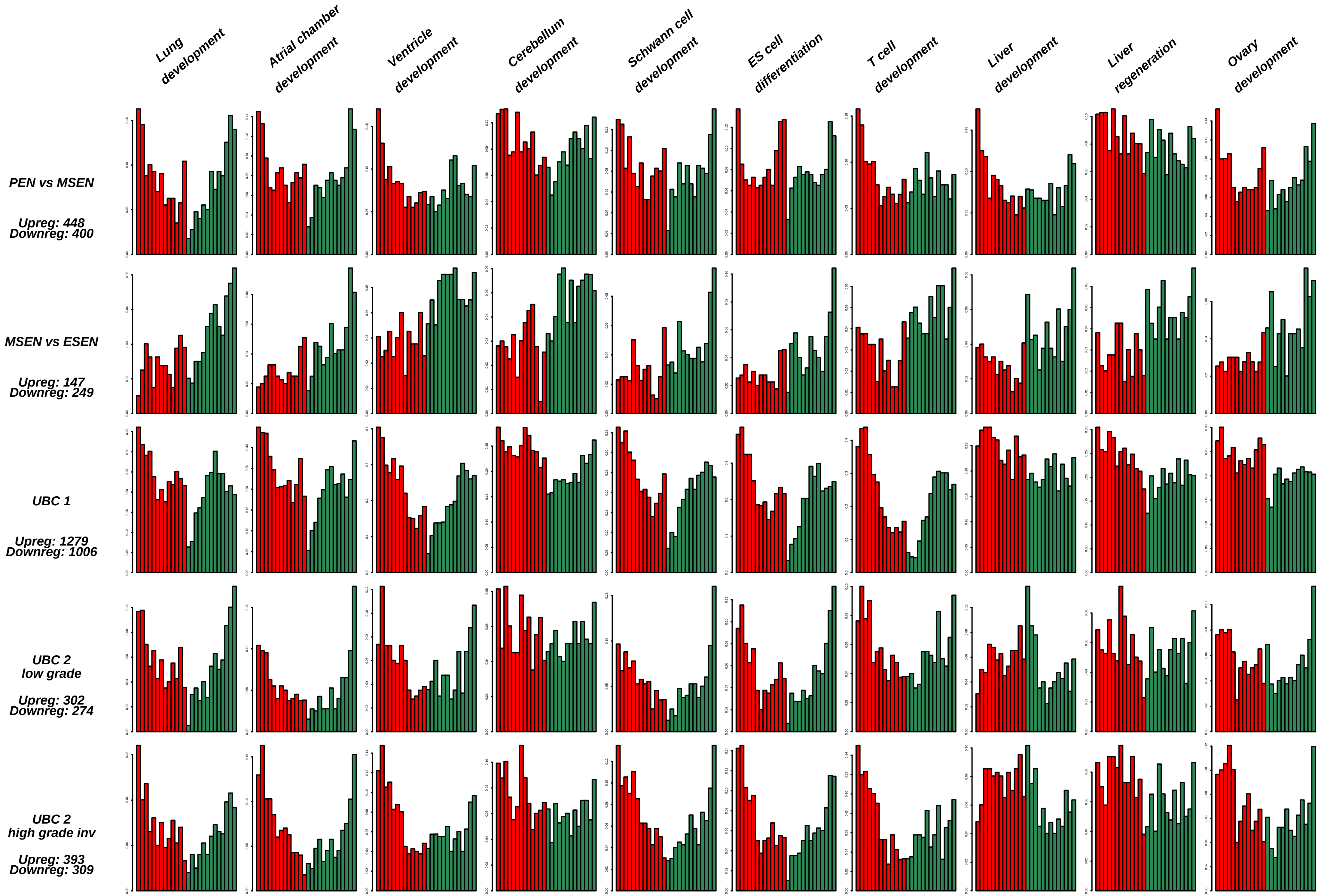

Lung development
Atrial chamber development
Ventricle development
Cerebellum development
Schwann cell development
ES cell differentiation
T cell development
Liver development
Liver regeneration
Ovary development
PEN vs MSEN
Upreg: 448
Downreg: 400
MSEN vs ESEN
Upreg: 147
Downreg: 249
UBC 1
Upreg: 1279
Downreg: 1006
UBC 2
low grade
Upreg: 302
Downreg: 274
UBC 2
high grade inv
Upreg: 393
Downreg: 309

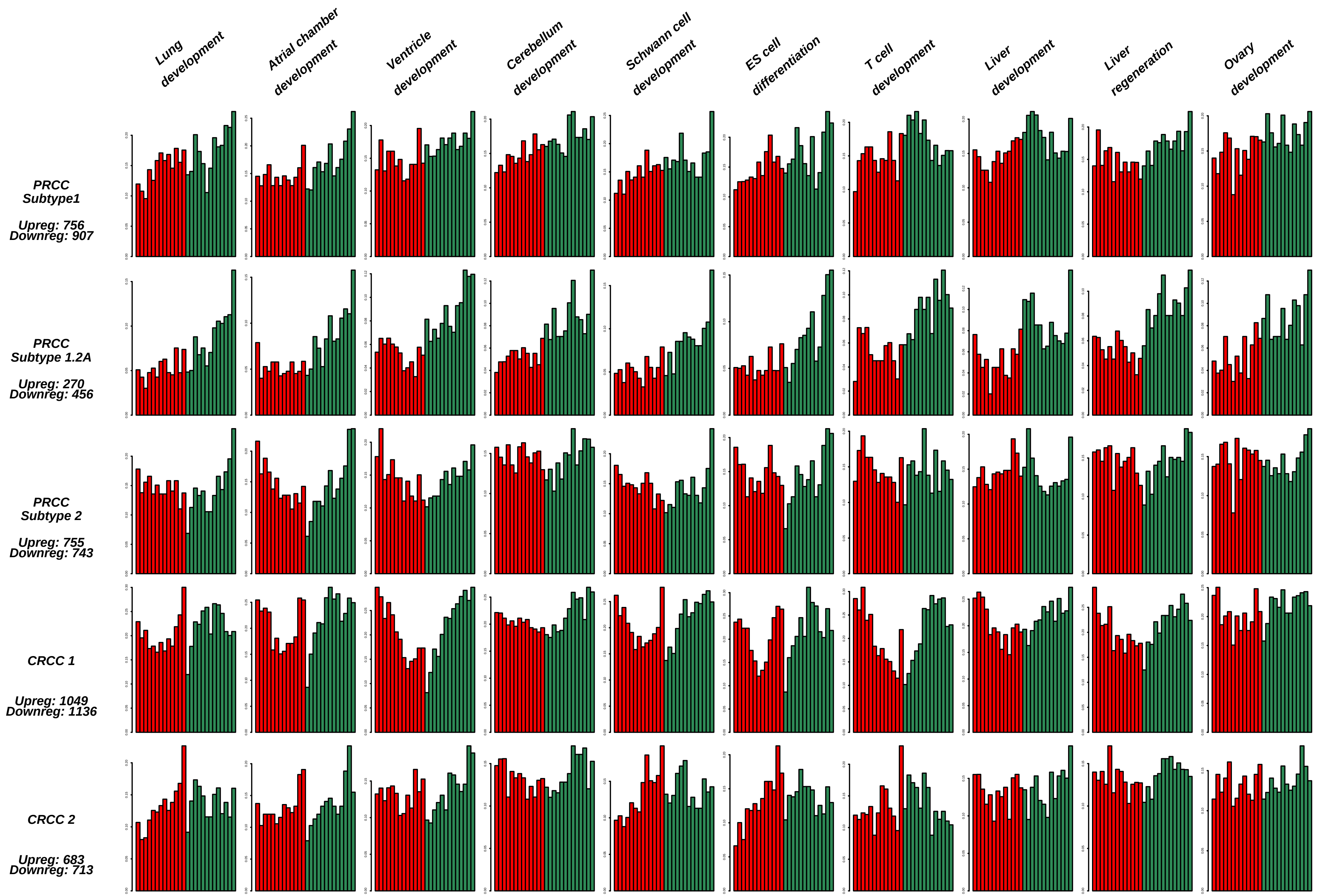

Lung development
Atrial chamber development
Ventricle development
Cerebellum development
Schwann cell development
ES cell differentiation
T cell development
Liver development
Liver regeneration
Ovary development
PRCC Subtype1
Upreg: 756
Downreg: 907
PRCC Subtype 1.2A
Upreg: 270
Downreg: 456
PRCC Subtype 2
Upreg: 755
Downreg: 743
CRCC 1
Upreg: 1049
Downreg: 1136
CRCC 2
Upreg: 683
Downreg: 713

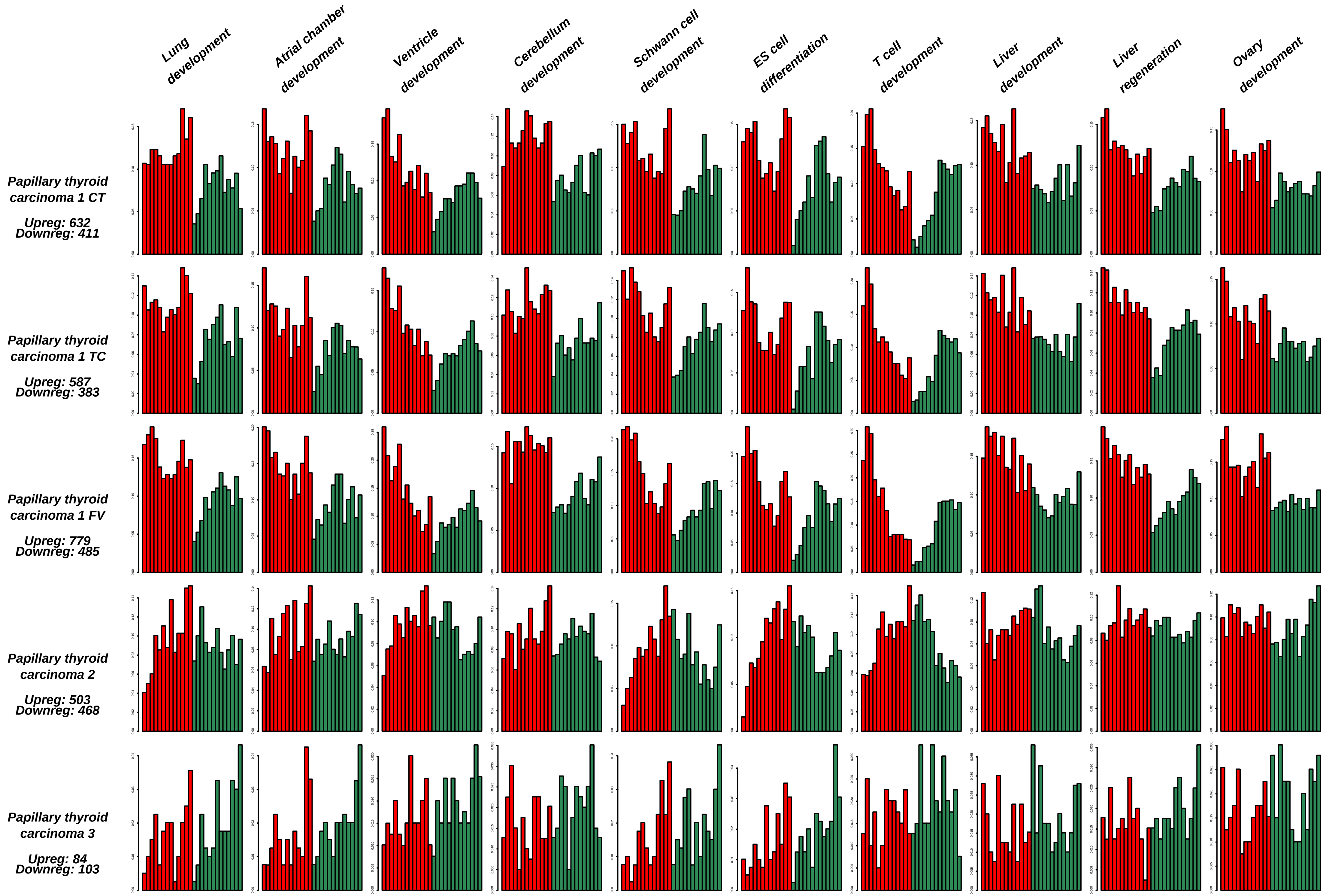
Lung development
Atrial chamber development
Ventricle development
Cerebellum development
Schwann cell development
ES cell differentiation
T cell development
Liver development
Liver regeneration
Ovary development
Papillary thyroid carcinoma 1 CT
Upreg: 632
Downreg: 411
Papillary thyroid carcinoma 1 TC
Upreg: 587
Downreg: 383
Papillary thyroid carcinoma 1 FV
Upreg: 779
Downreg: 485
Papillary thyroid carcinoma 2
Upreg: 503
Downreg: 468
Papillary thyroid carcinoma 3
Upreg: 84
Downreg: 103

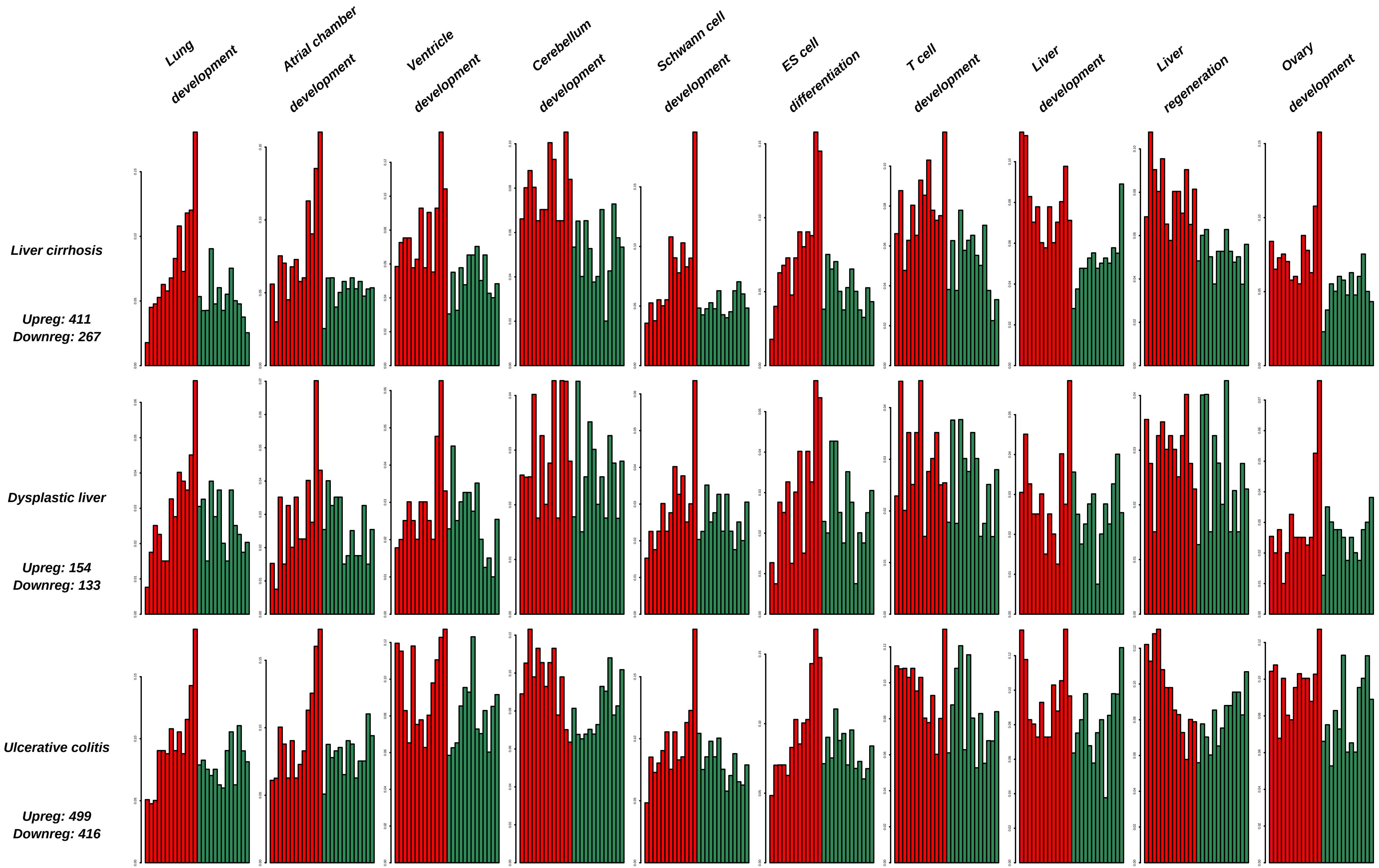

Lung development
Atrial chamber development
Ventricle development
Cerebellum development
Schwann cell development
ES cell differentiation
T cell development
Liver development
Liver regeneration
Ovary development
Liver cirrhosis
Upreg: 411
Downreg: 267
Dysplastic liver
Upreg: 154
Downreg: 133
Ulcerative colitis
Upreg: 499
Downreg: 416
