# Supplementary figures and images for: Analysis of gene expression in a developmental context emphasizes distinct biological leitmotifs in human cancers
Source: Genome Biol. 2008 Jul 8;9(7):R108. doi: 10.1186/gb-2008-9-7-r108 (PMC2530866; doi:10.1186/gb-2008-9-7-r108)

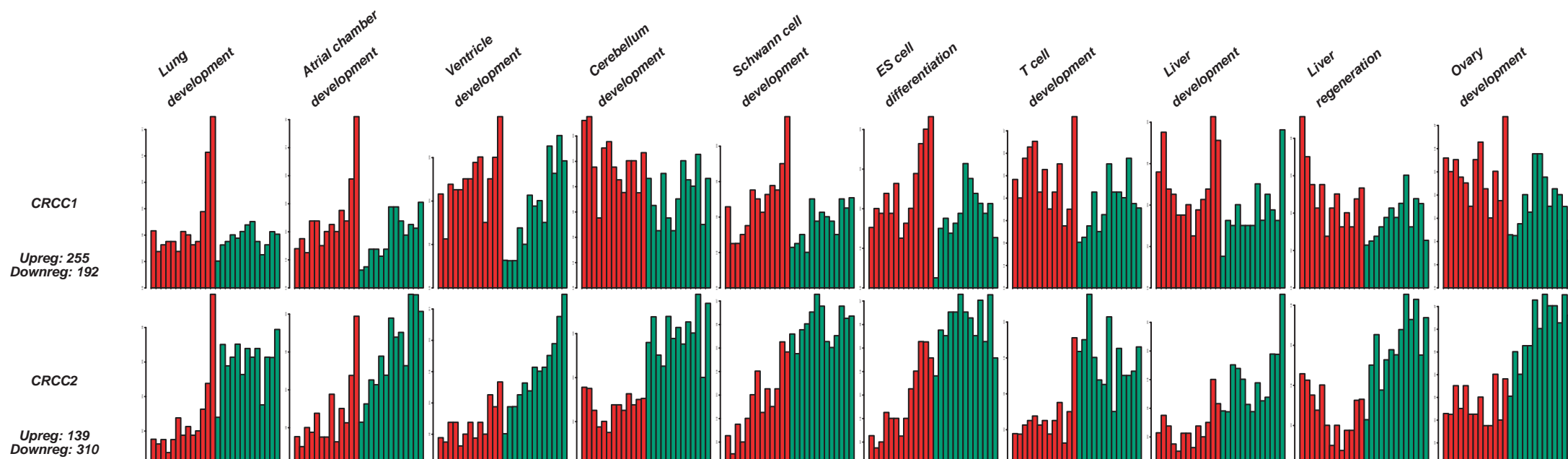

Supplement: Additional data file 3 — Frequency plots for the top 450 differentially expressed genes in CRCC1 and CRCC2. [file gb-2008-9-7-r108-S3.pdf]

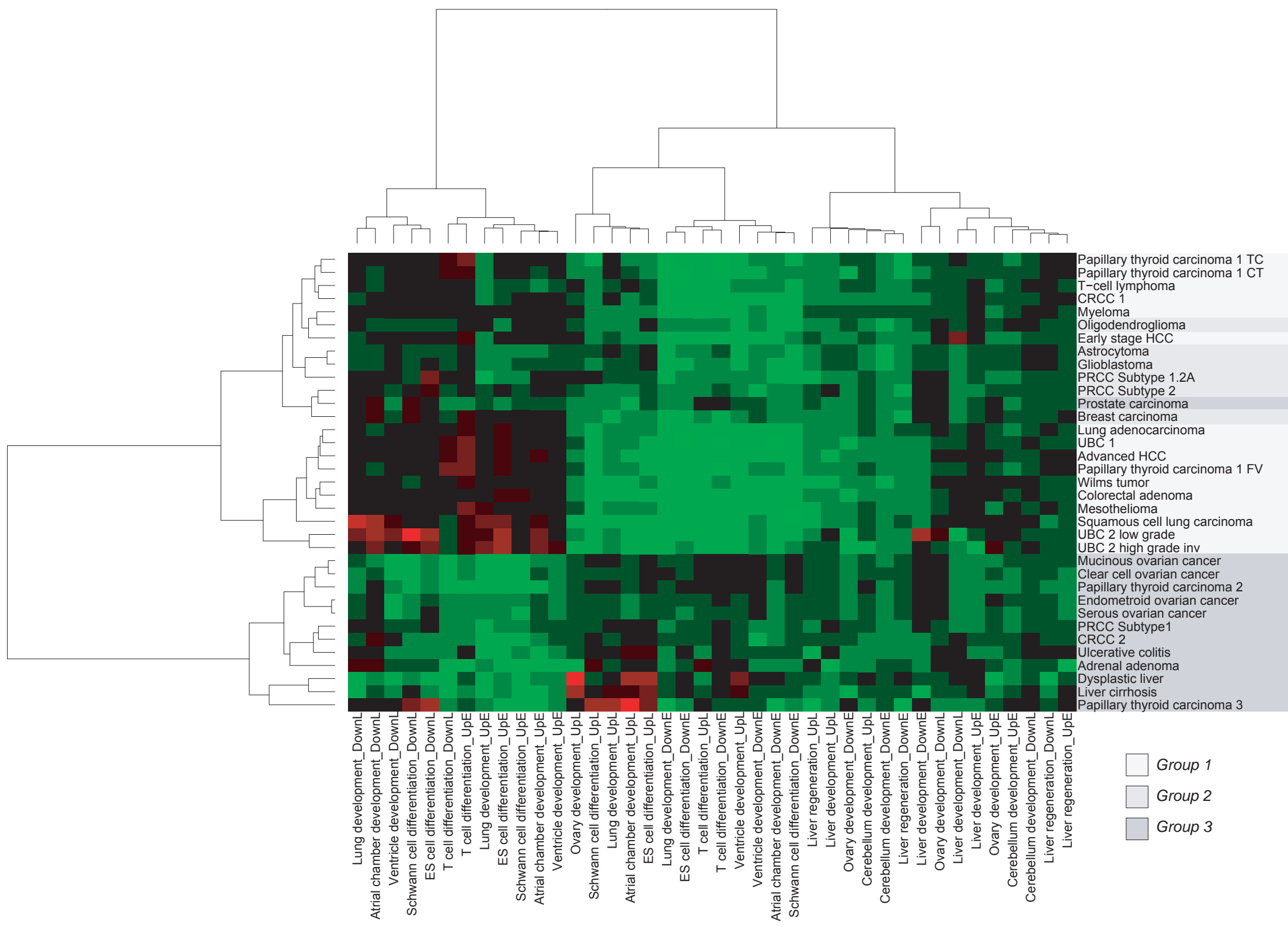

Supplement: Additional data file 4 — Heatmap of probability distribution slopes after CC subtraction (analogously to Figure 3). [file gb-2008-9-7-r108-S4.pdf]

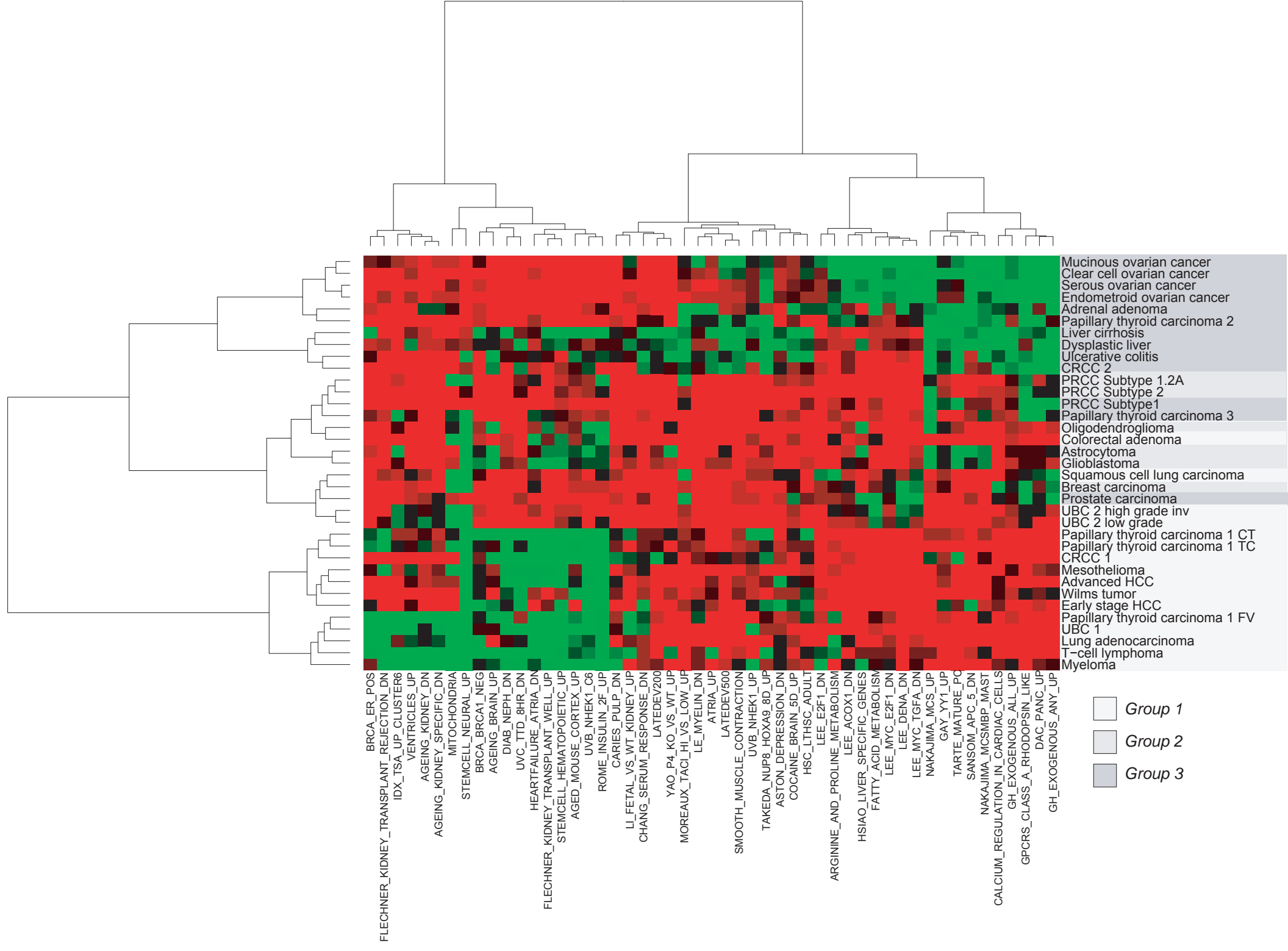

Supplement: Additional data file 5 — Heatmap of enrichment p-values for gene sets that ranked among the 20 most enriched in the downregulated genes of either group 1, 2 or 3. [file gb-2008-9-7-r108-S5.pdf]

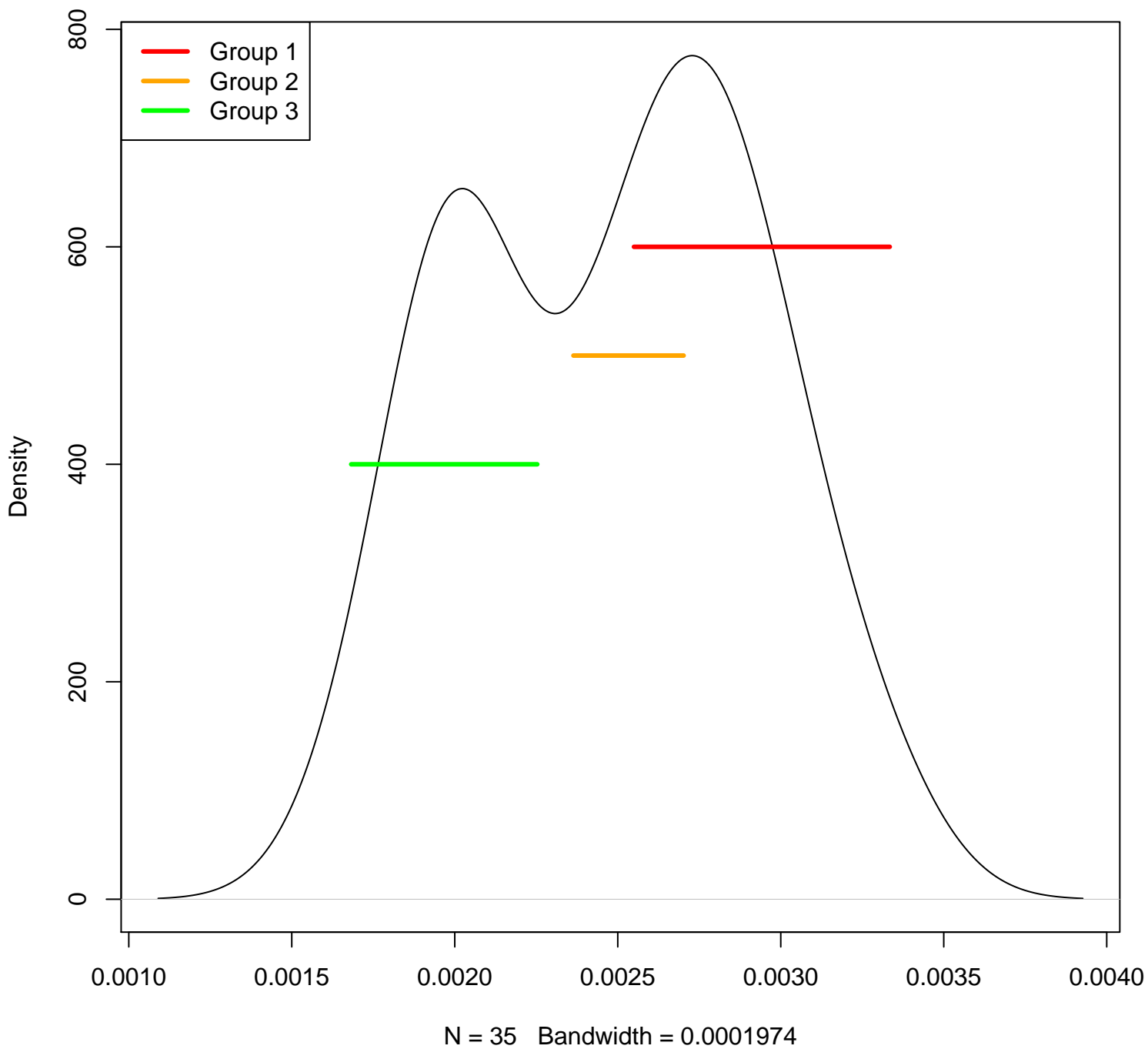

Supplement: Additional data file 8 — This shows a bimodal distribution with the left peak containing group 3 tumors, the right peak containing group 1 tumors and intermediate cases (group 2) falling in between. [file gb-2008-9-7-r108-S8.pdf]

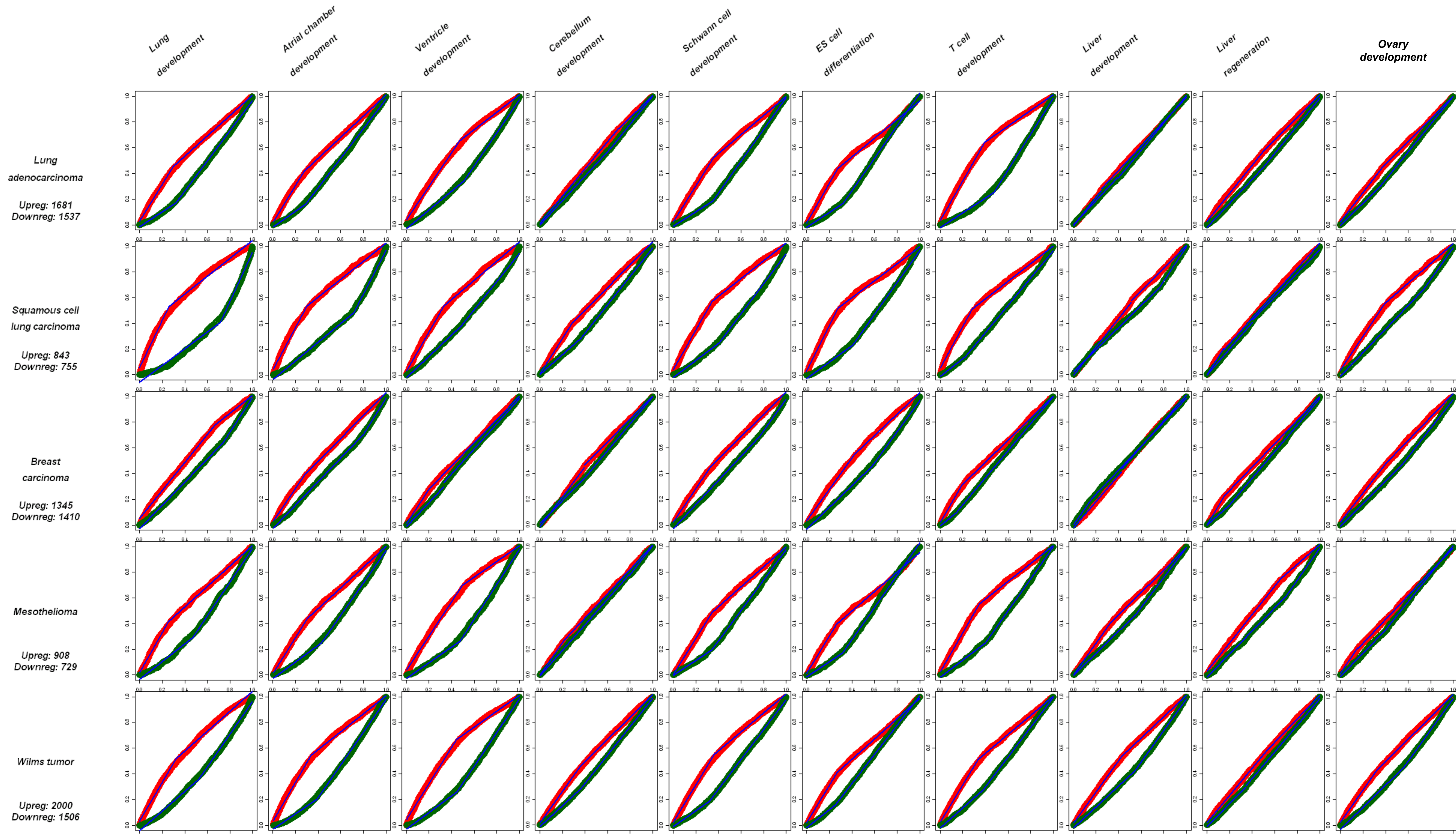

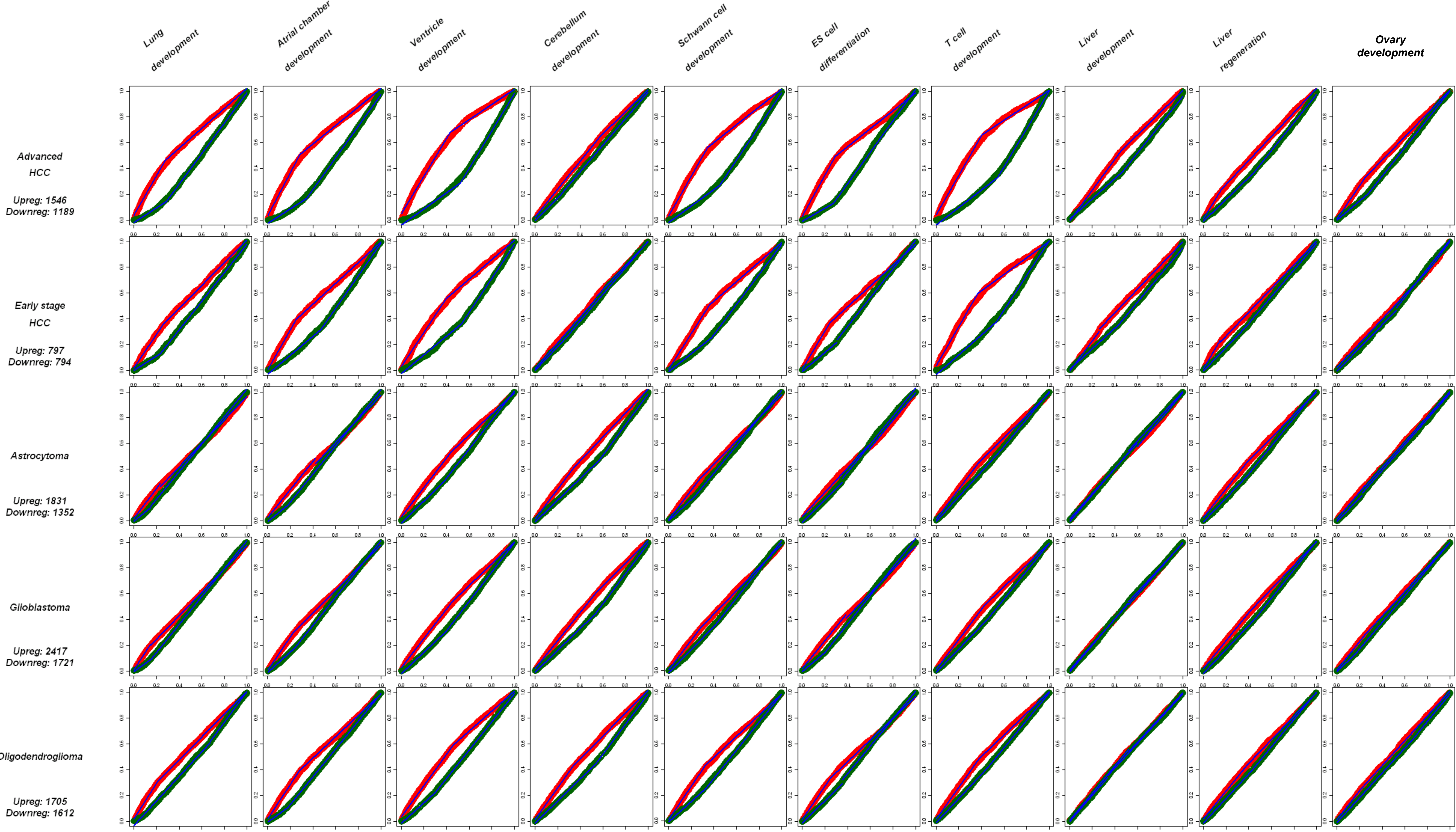

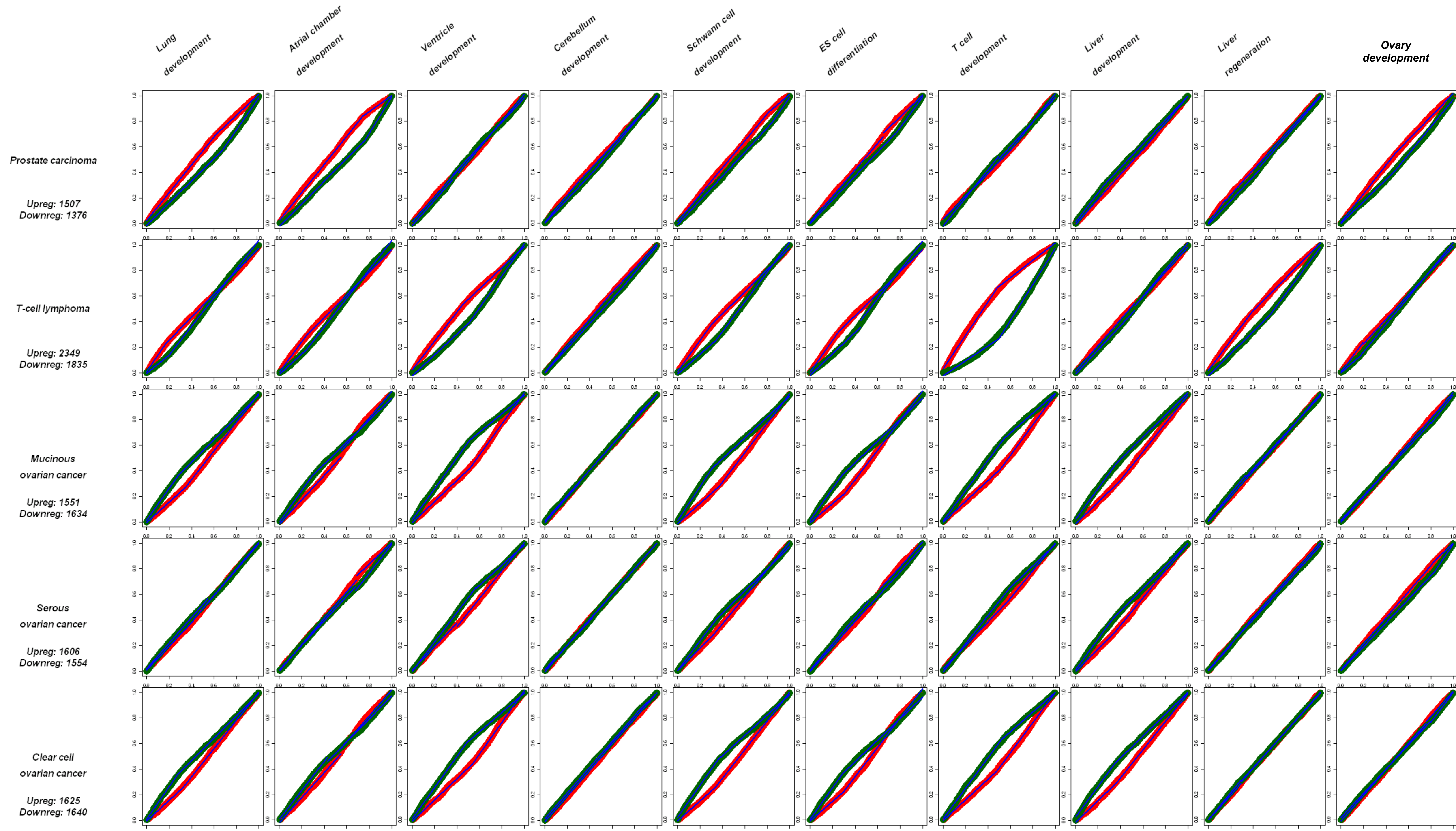

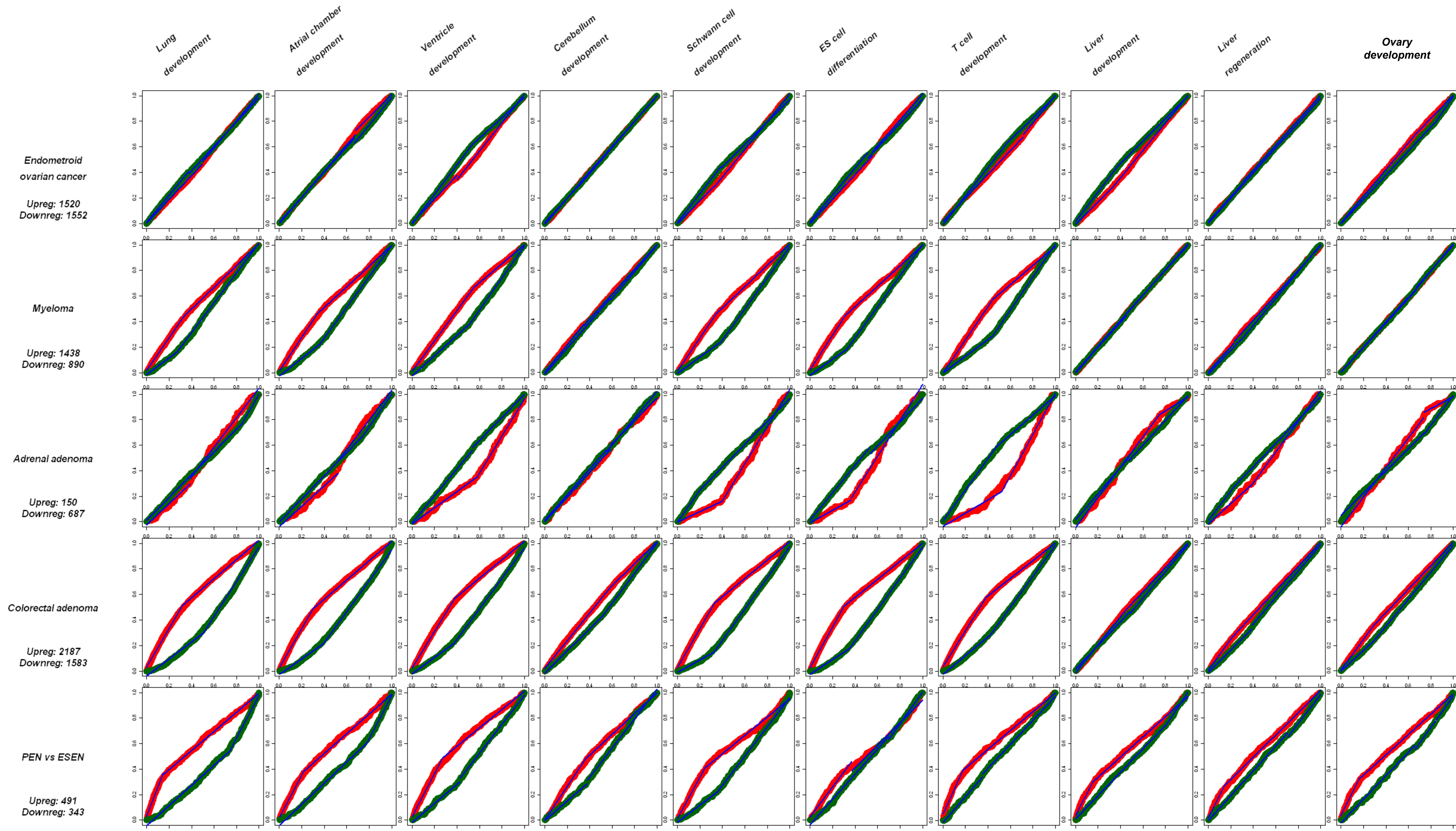

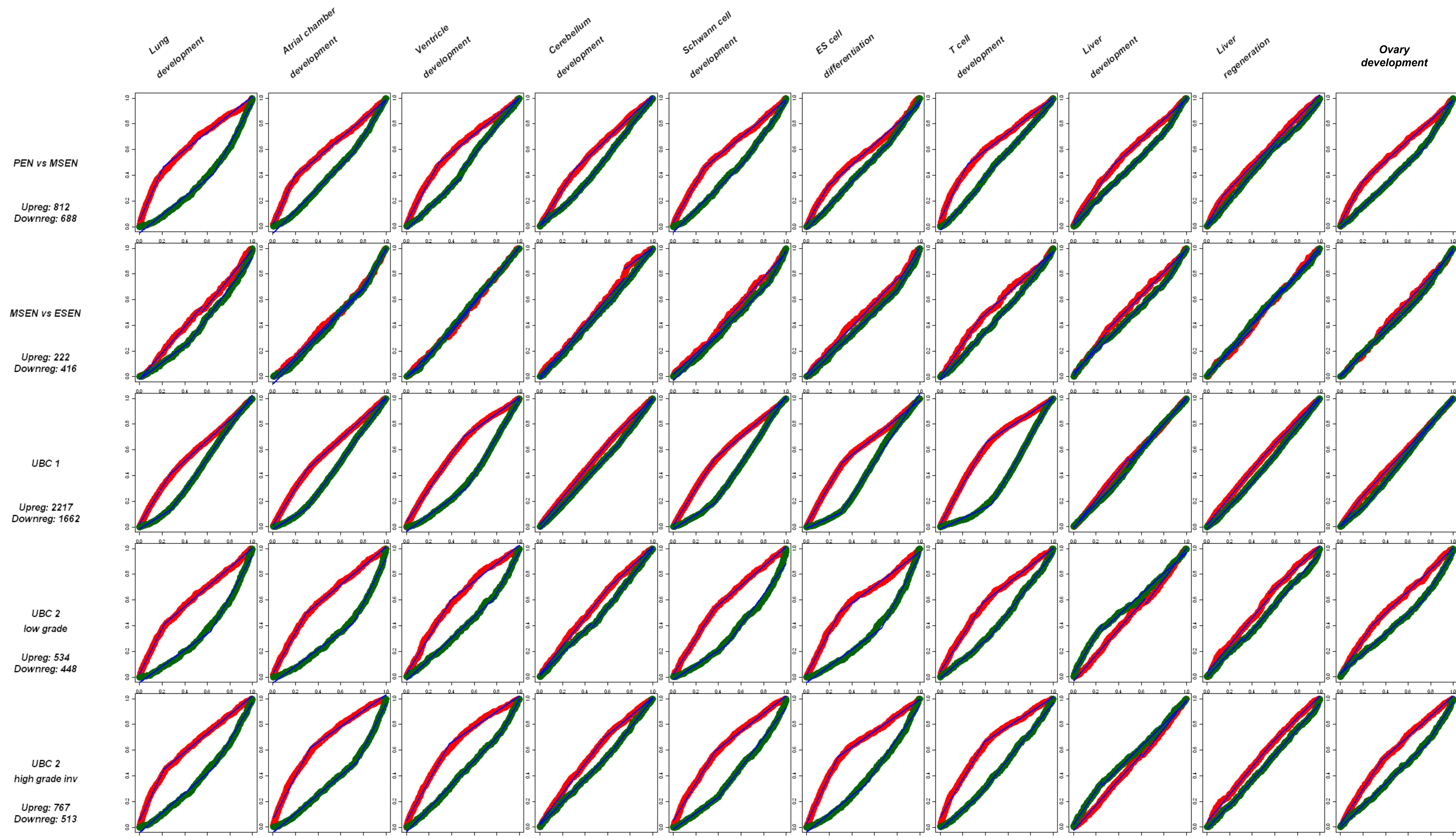

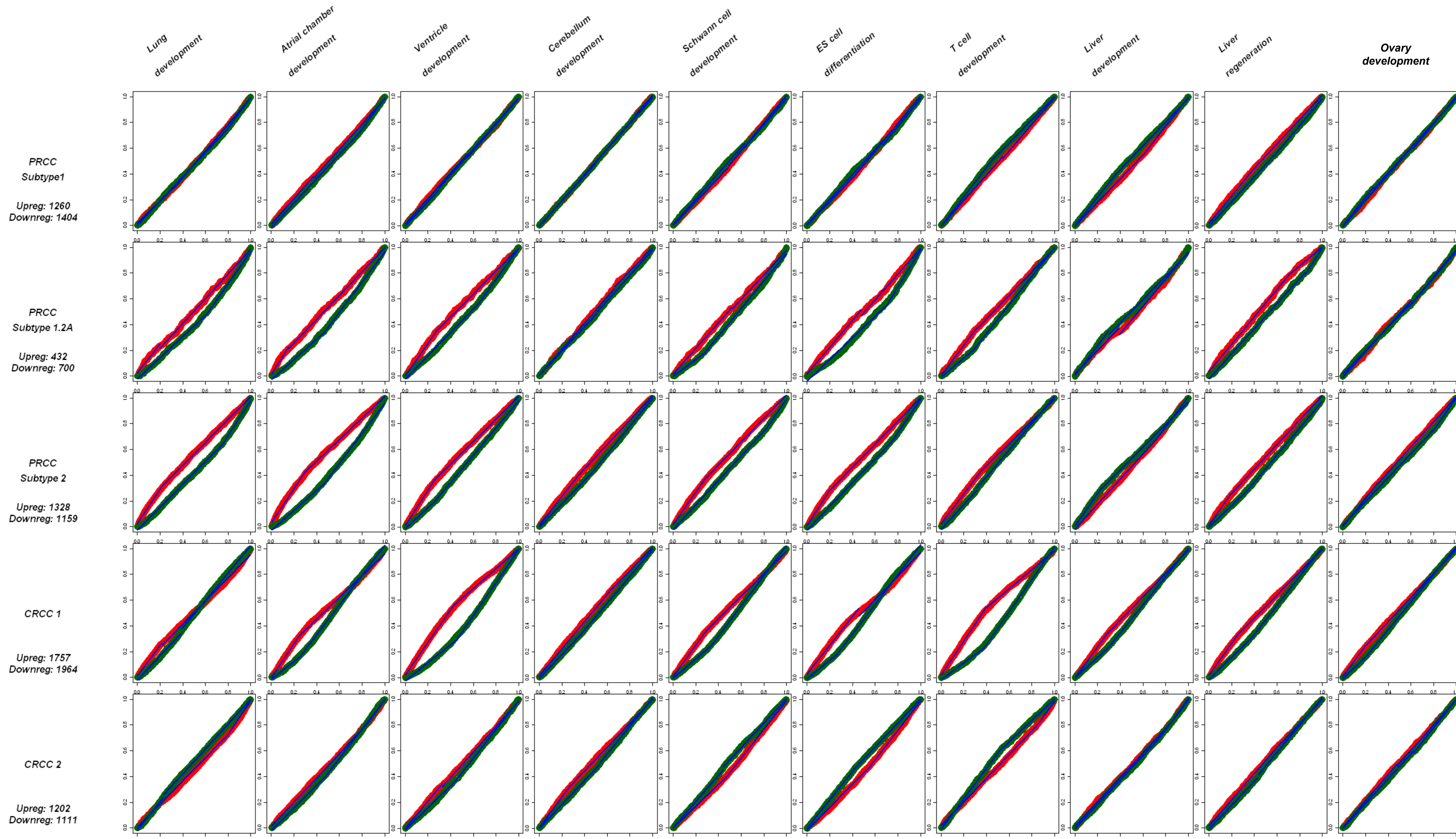

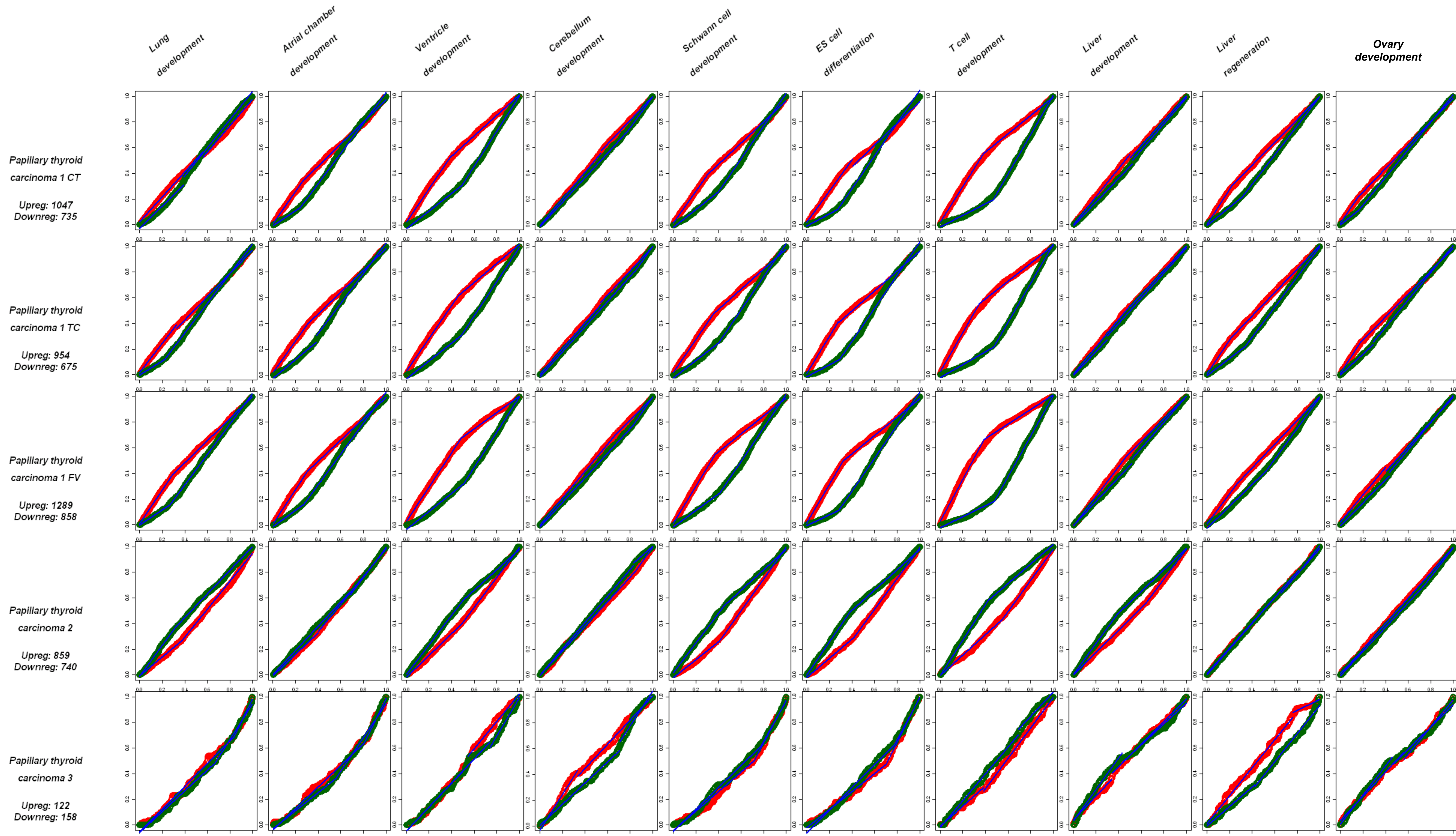

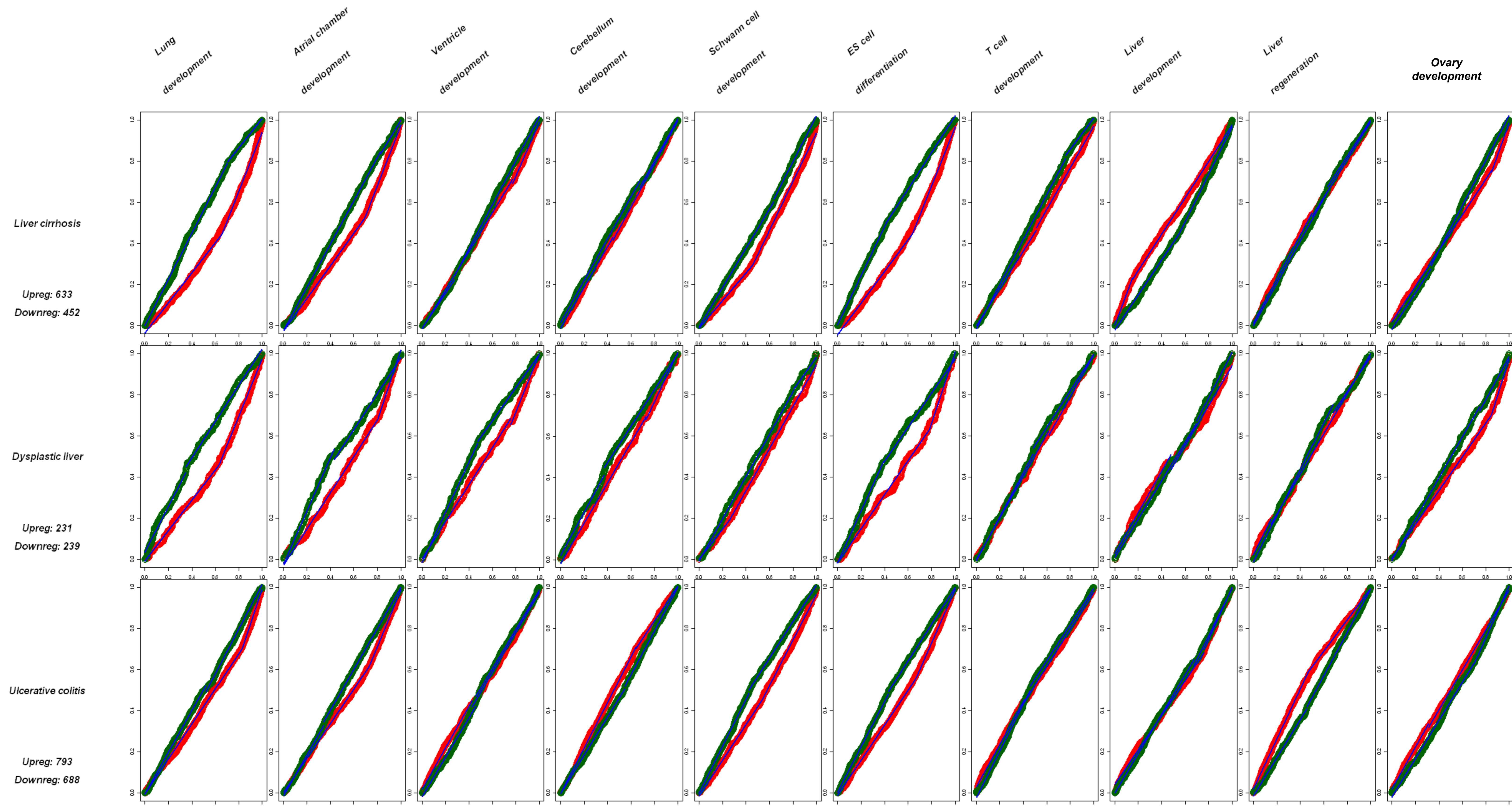

Supplement: Additional data file 9 — Probability distribution plots and linear regression fits for all cancers and all time series. [file gb-2008-9-7-r108-S9.pdf]

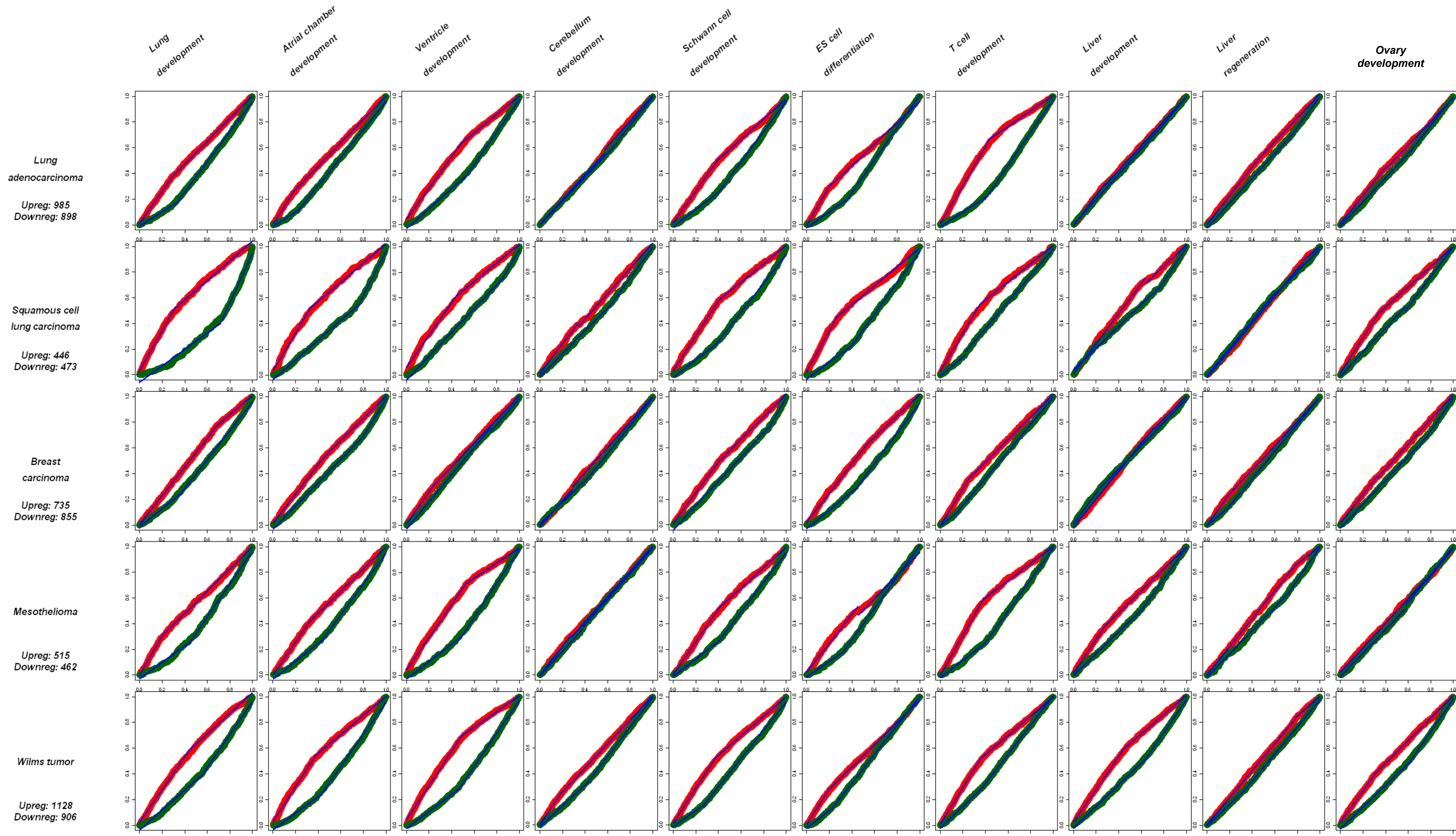

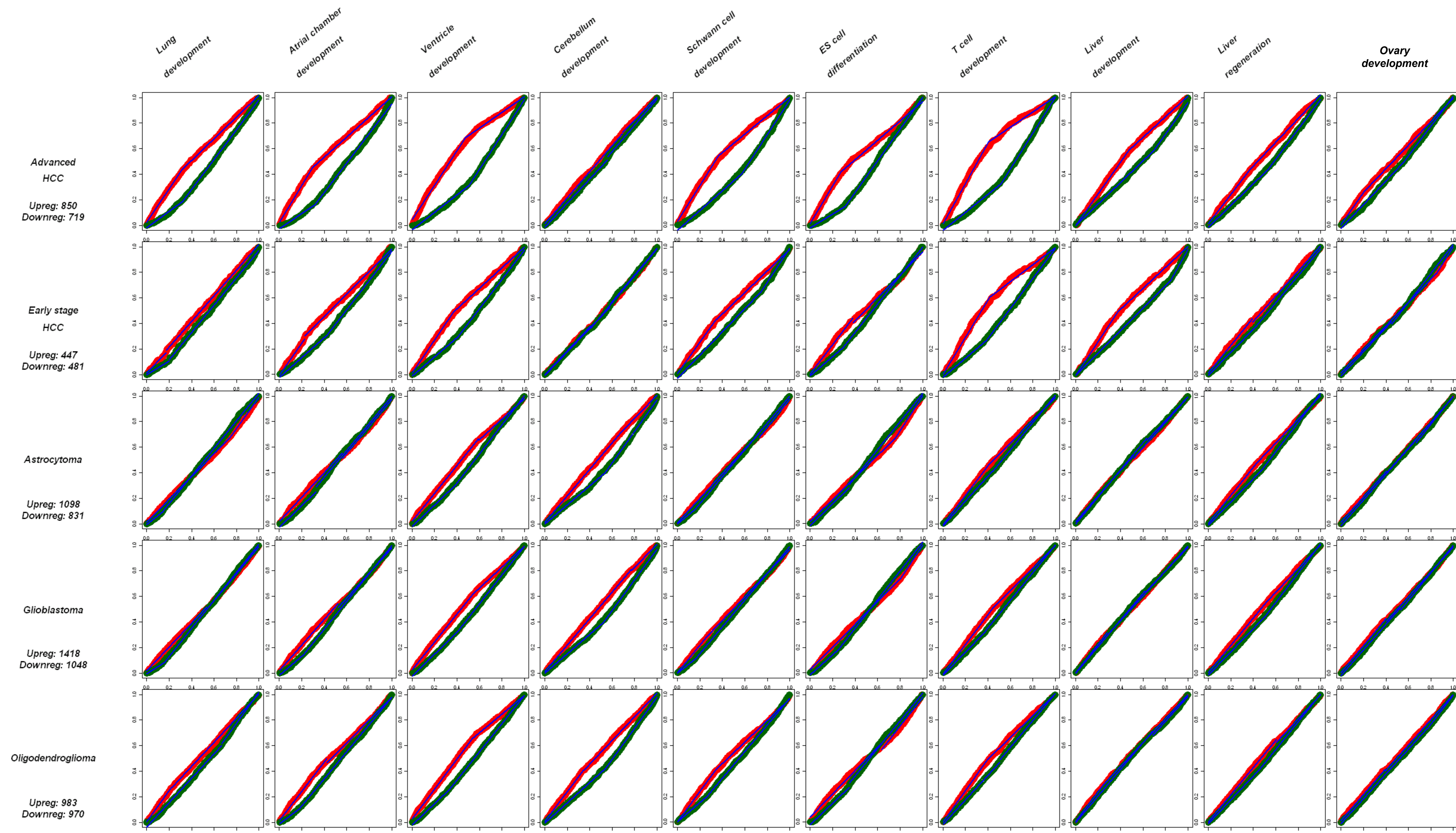

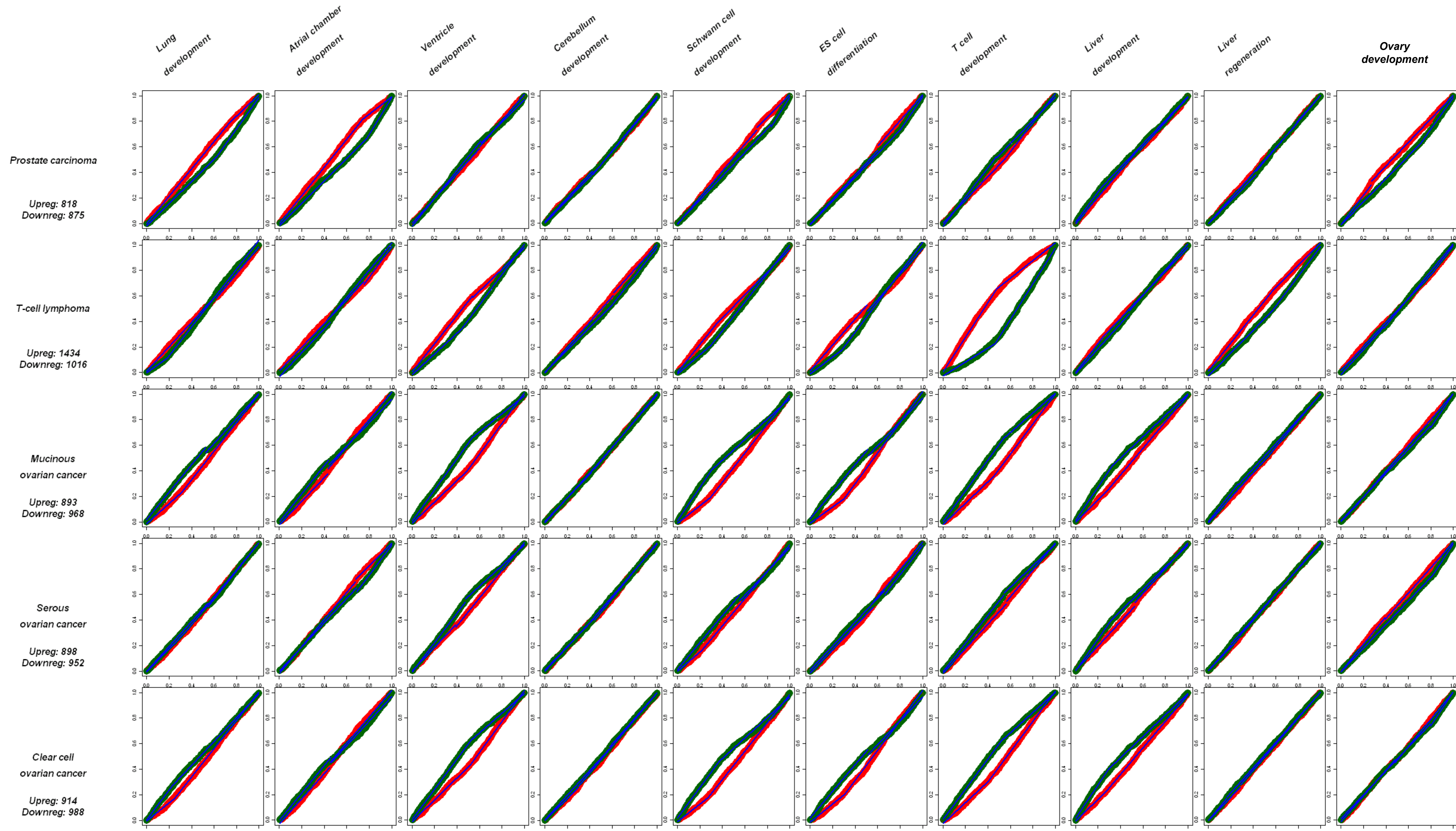

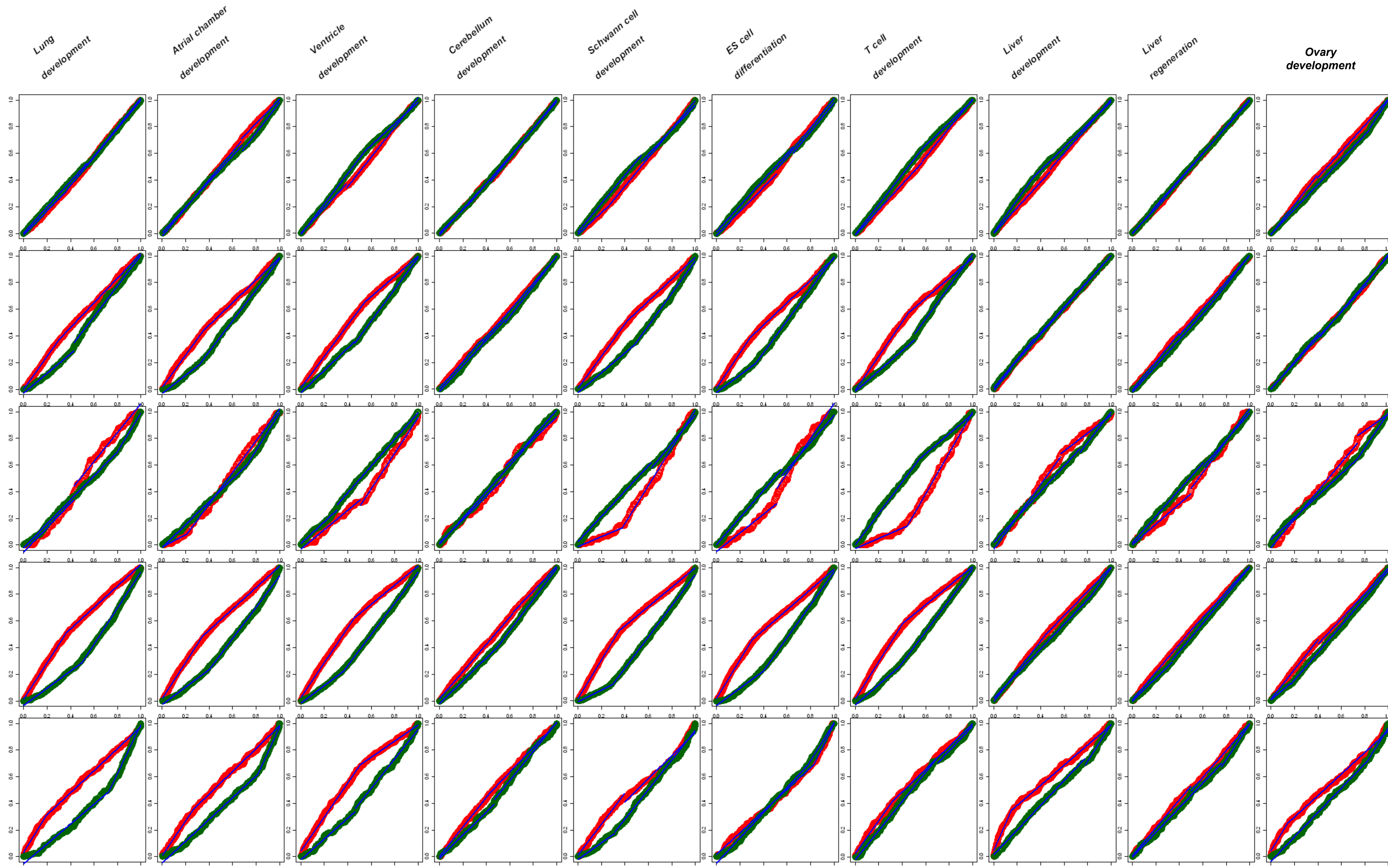

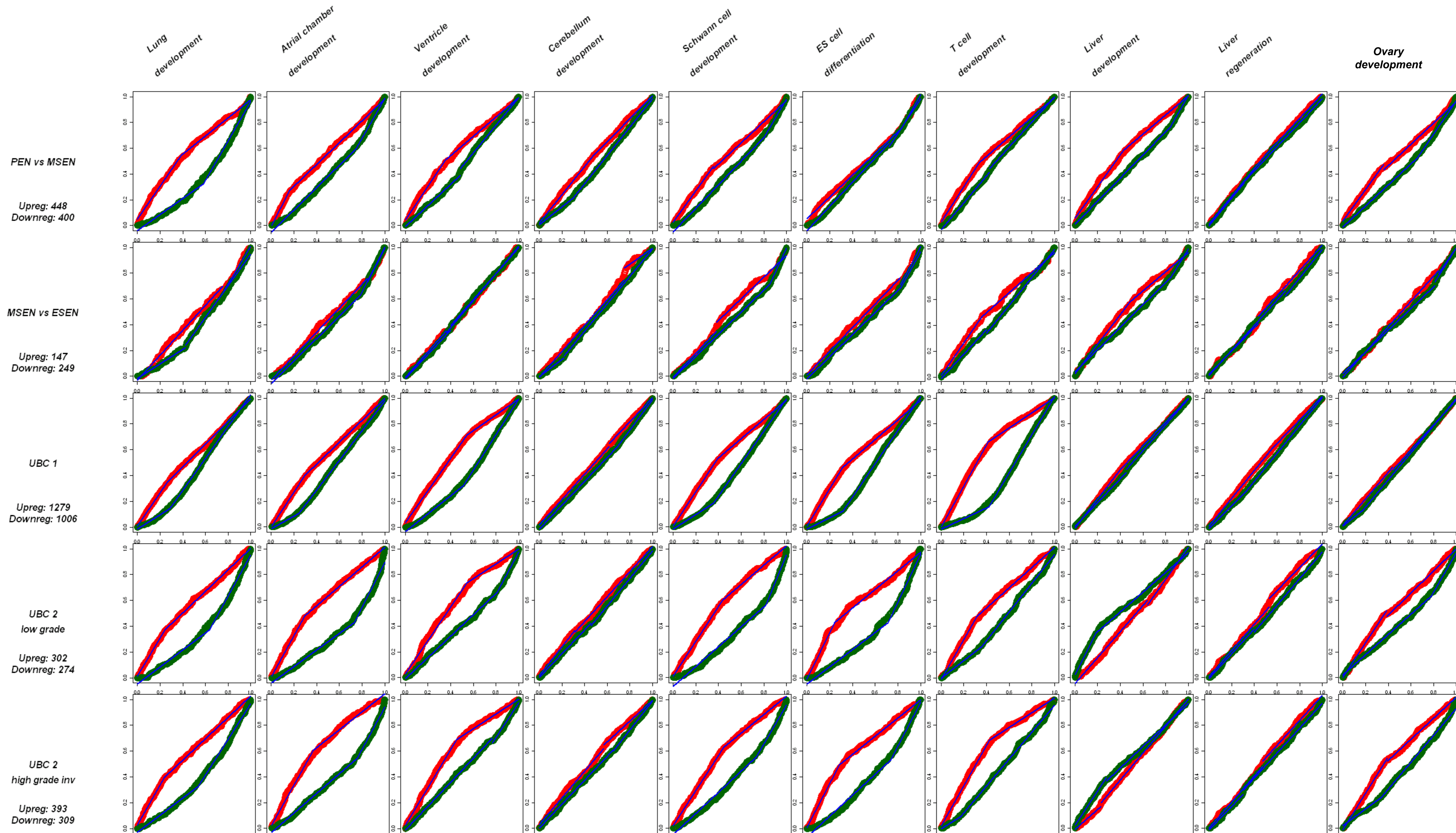

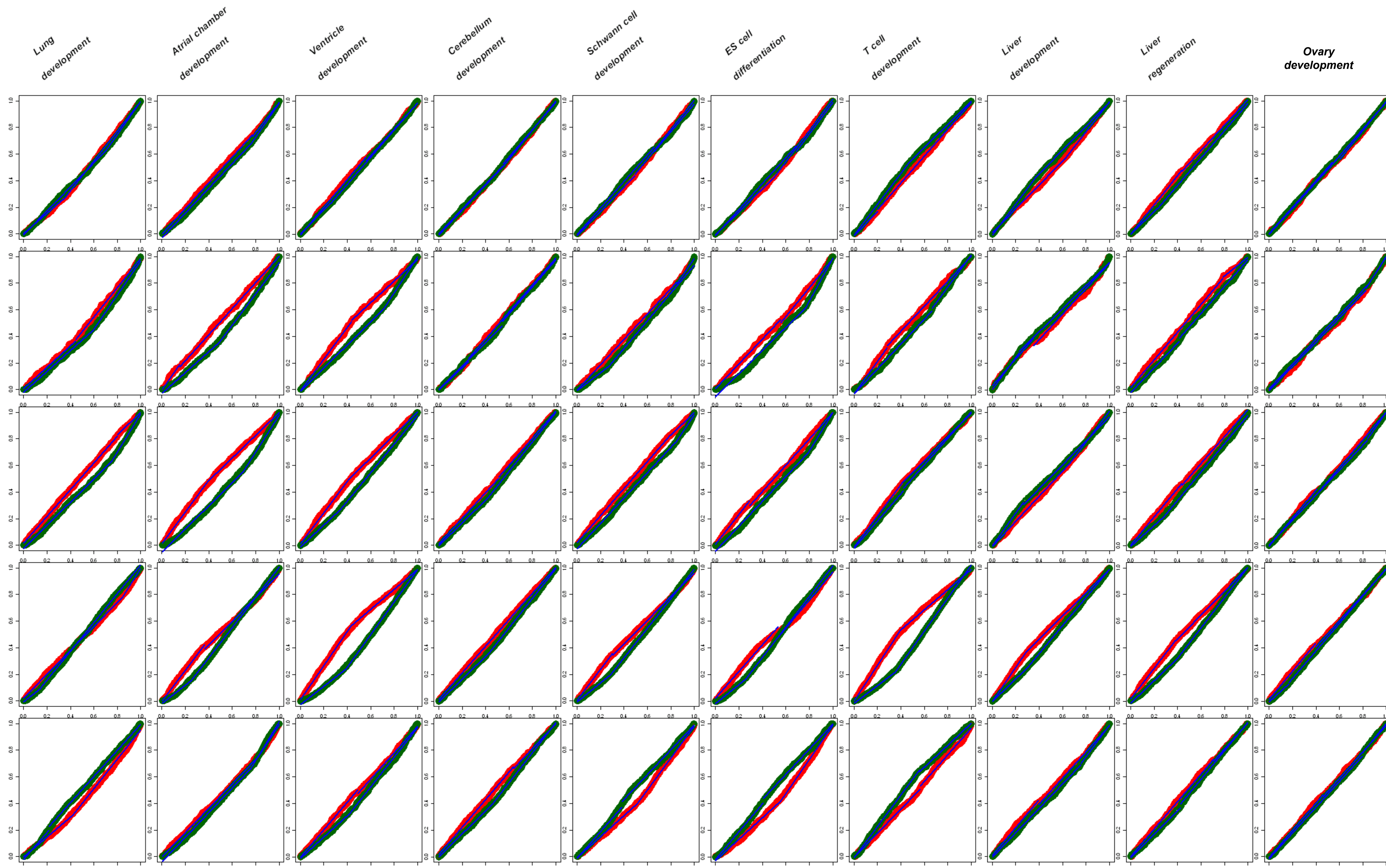

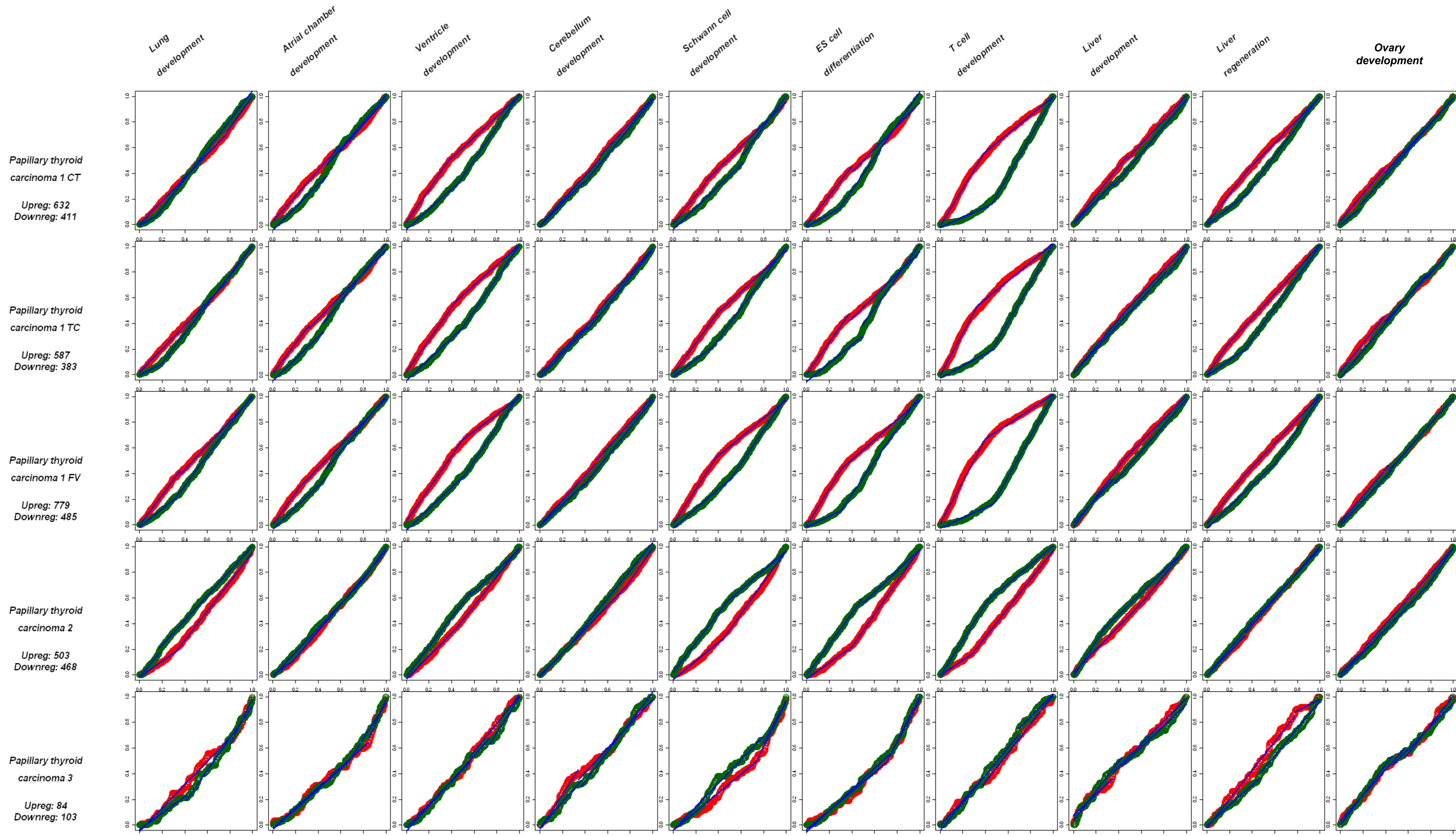

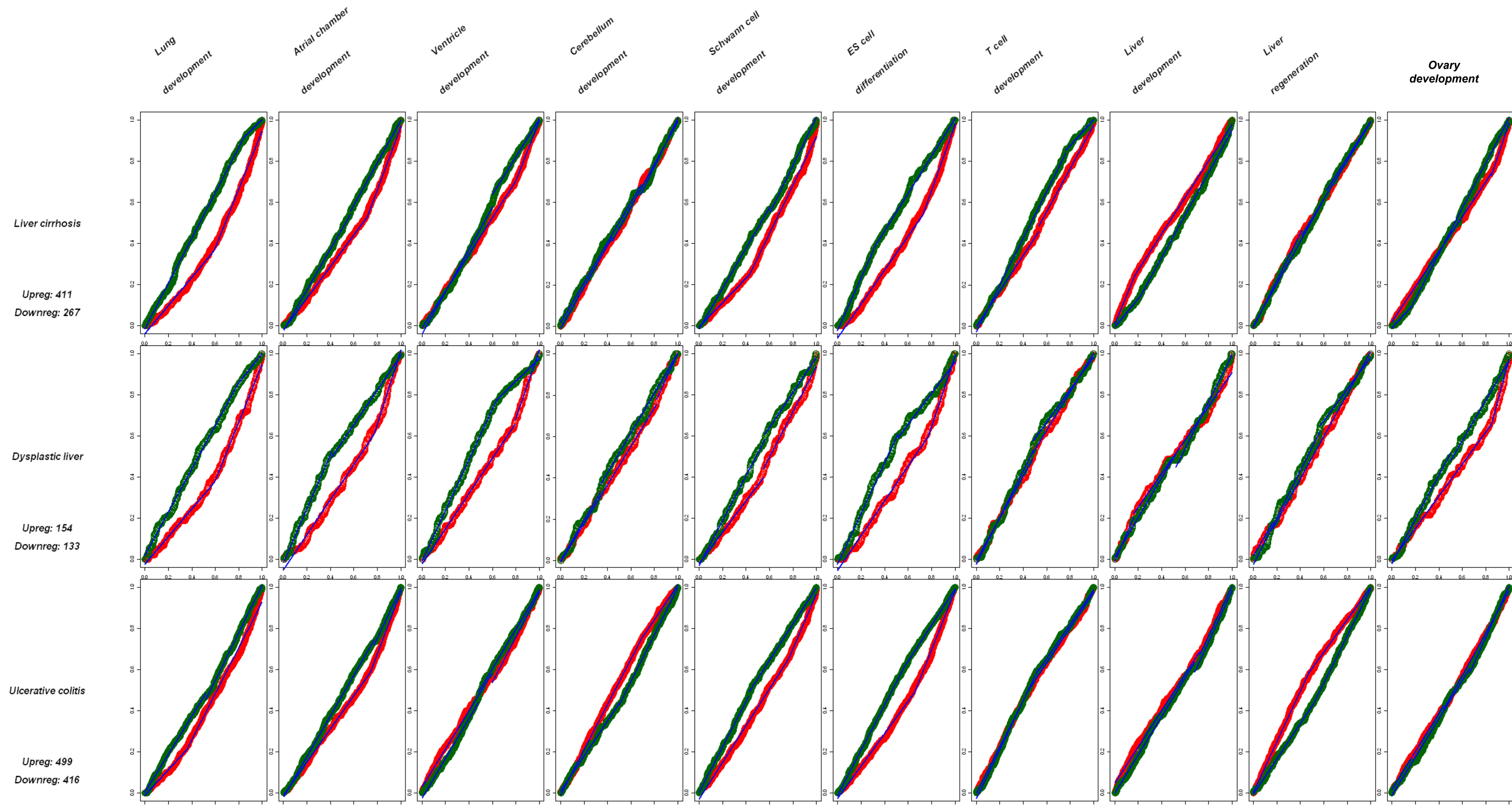

Supplement: Additional data file 10 — The same data as additional data file 9, but after CC subtraction. [file gb-2008-9-7-r108-S10.pdf]
